# Volatiles from the Psychrotolerant Bacterium *Chryseobacterium polytrichastri*

**DOI:** 10.1002/cbic.202000503

**Published:** 2020-09-16

**Authors:** Lukas Lauterbach, Jeroen S. Dickschat

**Affiliations:** ^1^ Kekulé Institute for Organic Chemistry and Biochemistry University of Bonn Gerhard-Domagk-Straße 1 53121 Bonn Germany

**Keywords:** fragmentation mechanisms, GC-MS, natural products, structure elucidation, volatile organic compounds

## Abstract

The flavobacterium *Chryseobacterium polytrichastri* was investigated for its volatile profile by use of a closed‐loop stripping apparatus (CLSA) and subsequent GC‐MS analysis. The analyses revealed a rich headspace extract with 71 identified compounds. Compound identification was based on a comparison to library mass spectra for known compounds and on a synthesis of authentic standards for unknowns. Important classes were phenylethyl amides and a series of corresponding imines and pyrroles.

## Graphical abstract

**Over 70** volatile organic compounds have been identified in a headspace extract from *C. polytrichastri*. The extract was particularly rich in nitrogen‐containing natural products, many of which were structurally derived from 2‐phenylethylamine. Several compounds are new natural products, their suggested structures are based on the observed mass spectral fragmentation, confirmed by synthesis of reference substances.

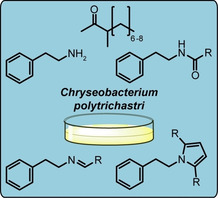

## 1 Introduction

The genus *Chryseobacterium* is the second largest within the family *Flavobacteriaceae* with more than 100 described members.^[1]^ The number of identified species has increased quickly over the last decades, from only seven species being known in 2002.^[2]^ *Chryseobacterium* spp. are present in various habitats of different environmental conditions including soil,^[3]^ Antarctic sea water,^[4]^ diseased fish^[5]^ and human tissue samples like for the pathogen *Chryseobacterium gleum*.^[6]^ Although the ecology and pathogenicity of chryseobacteria and phylogenetically closely related bacteria has been well investigated, their secondary metabolism so far remained disregarded. First studies include the isolation of sulfobacins A and B from *Chryseobacterium* sp. NR 2993,^[7]^ a report on volatile methyl ketones from bacteria of distinctly related genera isolated from arctic sea water,^[8]^ and the identification of two diterpene synthases from *Chryseobacterium polytrichastri* and *Chryseobacterium wanjuense* producing diterpenes with novel skeletons.^[9]^ *C. polytrichastri* DSM 26899 investigated in this study was isolated from the moss *Polytrichastrum formosum* which was collected from the Gawalong glacier in Tibet, China.^[10]^ Psychrotolerance has been reported within this genus before, the most astonishing example probably being *Chryseobacterium greenlandense* which was isolated from a 120 000‐year‐old layer of ice.^[11]^ Here we report on the volatile organic compounds (VOCs) emitted by *C. polytrichastri* and provide one of the first studies on the secondary metabolism within the genus *Chryseobacterium*.

## 2 Results and Discussion

**Figure 1:**
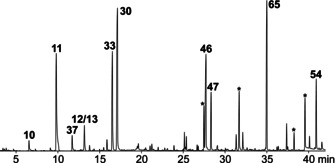
Total ion chromatogram of the headspace extract obtained from CLSA analysis of *C. polytrichastri*. Peaks marked with asterisks represent the plasticiser dibutyl phthalate and major unidentified aromatic compounds.

An agar‐plate culture of *C. polytrichastri* was subjected to a closed‐loop stripping apparatus (CLSA)^[12]^ and the volatiles were collected on charcoal filter traps for 24 hours. The charcoal filter was extracted with dichloromethane and the obtained headspace extract was analysed directly by GC‐MS. The total ion chromatogram (TIC) showed a rich bouquet consisting of 71 compounds identified by this study, originating from various compound classes (Figure 1, Figure S1 and Table S1 in the Supporting Information).

**Figure 2:**
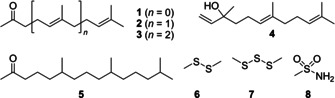
Structures of terpenoids and sulfur volatiles observed in the headspace extract of *C. polytrichastri*.

For several well‐known volatiles the identification by comparison of mass spectra to library spectra and of retention indices to previously published data was possible. Among terpenoids this included the nor‐carotenoids^[13]^ 6‐methylhept‐5‐en‐2‐one (**1**), geranyl (**2**) and farnesyl acetone (**3**), nerolidol (**4**), and the saturated derivative 6,10,14‐trimethylpentadecan‐2‐one (**5**). The widely distributed sulfur volatiles dimethyl disulfide (**6**) and dimethyl trisulfide (**7**) and the less frequently observed methanesulfonamide (**8**) were also identified (Figure 2).[^14^, ^15^]

**Figure 3:**
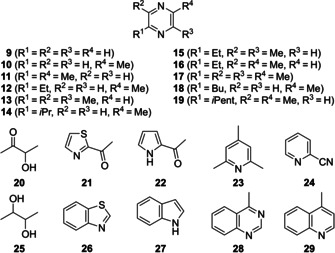
Structures of heteroaromatic compounds found in the headspace extract of *C. polytrichastri*.

The most abundant compound class were nitrogen‐containing volatiles, including pyrazine (**9**) and a series of alkylated derivatives (**10**–**19**, Figure 3), altogether making up around 13 % of the headspace extract. Pyrazines with diverse substitution patterns are known as headspace constituents from many fungi and bacteria,[^14^, ^15^, ^16^] and a biosynthetic pathway for alkylated compounds via acetoin (**20**) has been discovered by a combined labelling and mutation study in *Corynebacterium glutamicum*.^[17]^ This biosynthetic origin is also plausible for *C. polytrichastri* since butane‐2,3‐diol (**25**), potentially arising by reduction of **20**, is present in the headspace extract. Alternatively, pyrazines with branched groups such as **14** might arise by dimerisation of amino acids or amino aldehydes.[^18^, ^19^]

**Figure 4:**
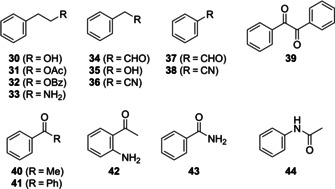
Structures of aromatic compounds contributing to the headspace extract of *C. polytrichastri*.

Other heteroaromatic nitrogen compounds were found in traces, represented by 2‐acetylthiazole (**21**), 2‐acetylpyrrole (**22**), the pyridine derivatives 2,4,6‐trimethylpyridine (**23**) and 2‐cyanopyridine (**24**), and the four bicyclic compounds benzothiazole (**26**), indole (**27**), 4‐methylquinazoline (**28**) and 4‐methylquinoline (**29**; Figure 3). Aromatic compounds include the main compound 2‐phenylethanol (**30**, 13.6 %), well‐known from several bacteria,^[15]^ and some of its derivatives such as phenylacetaldehyde (**34**), the acetate (**31**) and benzoate esters (**32**). Further compounds were benzyl alcohol (**35**), that has previously been observed in other *Flavobacteriaceae*,^[4]^ benzaldehyde (**37**) and diphenylethanedione (**39**), acetophenone (**40**), benzophenone (**41**) and 2‐aminoacetophenone (**42**). The latter might be a precursor for **29** that is a formal condensation product of **42** with acetaldehyde. Intriguingly, the combination of both compounds was previously observed in *Myxococcus xanthus* and *Streptomyces caviscabies*.[^20^, ^21^] Additionally, the two amides benzamide (**43**), *N*‐phenylacetamide (**44**), and two nitriles, benzonitrile (**38**) and phenylacetonitrile (**36**), were observed as minor constituents (Figure 4).

**Scheme 1:**
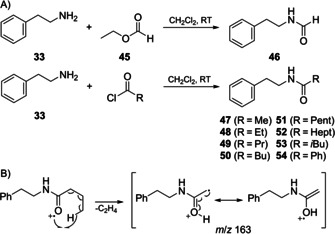
A) Synthesis of *N*‐(2‐phenylethyl)amides. B) McLafferty rearrangement from **49** leading to *m*/*z* 163.

**Figure 5:**
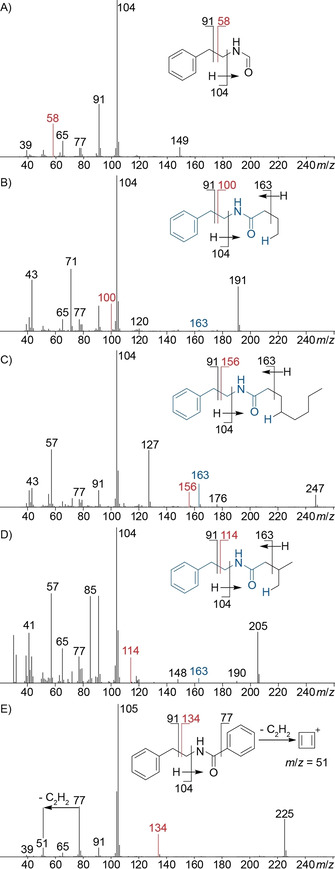
EI‐MS spectra of A) *N*‐(2‐phenylethyl)formamide (**46**), B) *N*‐(2‐phenylethyl)butanamide (**49**), C) *N*‐(2‐phenylethyl)octanamide (**52**), D) *N*‐(2‐phenylethyl)‐3‐methylbutanamide (**53**) and E) *N*‐(2‐phenylethyl)benzamide (**54**). Red indicates ions arising from benzyl group cleavage, blue indicates fragments originating from McLafferty rearrangement.

2‐Phenylethylamine (**33**) was also found, while several of its derivatives could not be identified simply from their mass spectra, since no good hits to library spectra were conceived. Therefore, structural suggestions were made based on the fragment ions observed in the EI mass spectra. One additional compound included in our MS library was *N*‐(2‐phenylethyl)formamide (**46**), while no literature retention index for this compound was available. A synthesis from ethyl formate (**45**) and **33** (Scheme 1)^[22]^ confirmed the identity of the synthetic compound with the natural product. With this as a starting point a homologous series of 2‐phenylethyl amides (**47**–**52**), was suspected from their mass spectra, all showing a similar fragmentation pattern to the pattern reported for *N*‐(2‐phenylethyl)amides before,^[23]^ with a characteristic base peak at *m*/*z* 104 and molecular ions from *m*/*z* 149 for **46** increasing stepwise by 14 Da to *m*/*z* 247 for **52** (Figures 5 and S2). A second important fragment ion indicative of the chain length arising by cleavage of the benzyl group was observed from *m*/*z* 58 increasing to *m*/*z* 156 for **52**. Taken together, these data suggested the compounds **46**–**52** to represent a series of *N*‐(2‐phenylethyl)amides from formic to octanoic acid. A synthesis starting from **42** and the acid chlorides (Scheme 1A) confirmed this hypothesis.

According to the molecular ion **53** was an isomer of **50**, but eluted earlier from the GC, in agreement with a branched acid portion. The non‐branched amides with longer alkyl chains exhibited diagnostic fragment ions arising by McLafferty rearrangement with cleavage of the acid side chain (Scheme 1B, highlighted in blue in Figures 5 and S2).^[24]^ The corresponding McLafferty ion of **53** was observed at *m*/*z* 163, indicating an isovalerate portion, while the 2‐methylbutyrate derivative would require *m*/*z* 177. The structural proposal of *N*‐(2‐phenylethyl)‐3‐methylbutanamide for **53** was confirmed by synthesis (Scheme 1A).^[25]^ Furthermore, the structure of *N*‐(2‐phenylethyl)benzamide for **54** was identified from its mass spectrum (Figure 5E), whose molecular ion indicated an acid side chain with four additional degrees of unsaturation. Also the enhanced fragment ions at *m*/*z* 77 and 51 pointed to a phenyl group as in the benzoate derivative. The structure of *N*‐(2‐phenylethyl)benzamide for **54** was confirmed by synthesis of reference material. Compound **48** was previously isolated from the limnic bacterium *Bacillus* sp. GW90a,^[26]^ while **51**–**54** were reported before from *Xenorhabdus doucetiae*.^[25]^ Compound **54** is known as a moderate inhibitor of *N*‐acylhomoserine lactone sensors in *Escherichia coli* MT102 and *Pseudomonas putida* F117.^[27]^

**Figure 6:**
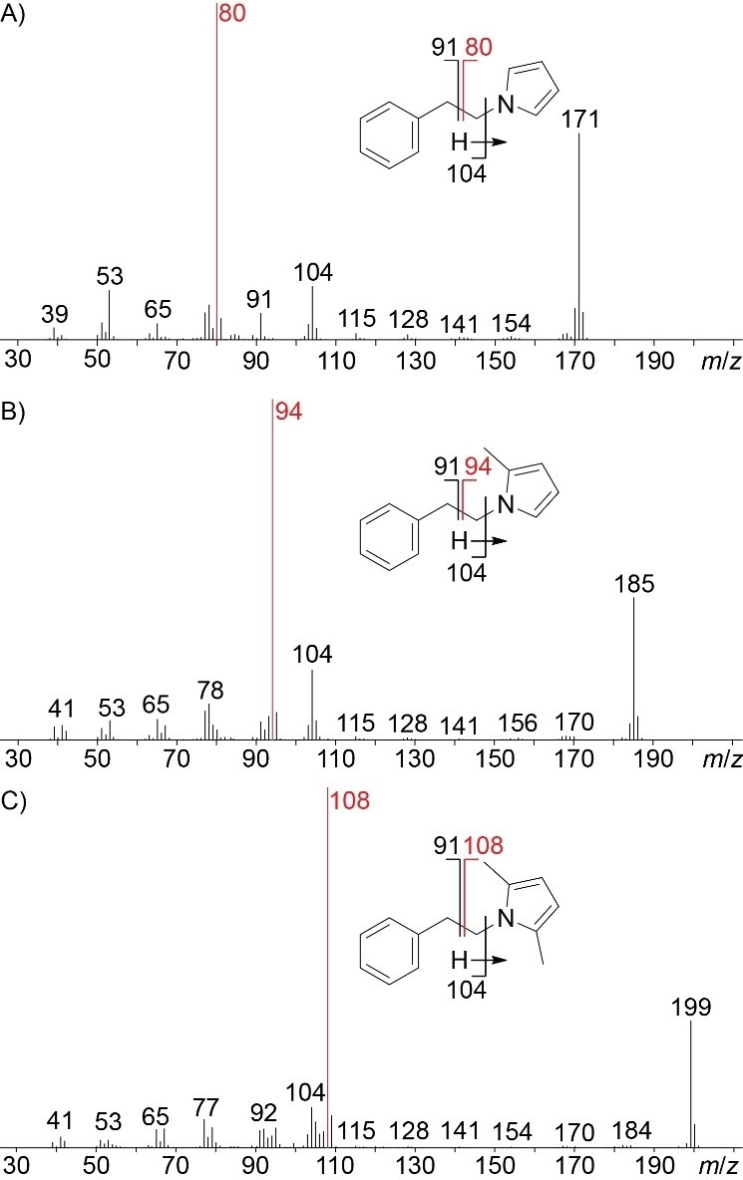
EI‐MS spectra of A) *N*‐(2‐phenylethyl)pyrrole (**56**), B) 2‐methyl‐*N*‐(2‐phenylethyl)pyrrole (**58**) and C) 2,5‐dimethyl‐*N*‐(2‐phenylethyl)pyrrole (**60**). Red indicates a fragment ion arising from benzyl group cleavage.

**Scheme 2:**
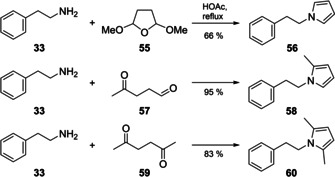
Synthesis of *N*‐(2‐phenylethyl)pyrroles.

The odd molecular ions for **56**, **58** and **60** with mass spectra not included in our libraries also pointed to nitrogen‐containing compounds (Figure 6). All three compounds showed fragment ions at *m*/*z* 104 and 91 and further typical fragment ions of aromatic compounds (*m*/*z* 77, 65 and 39), suggesting they might likewise contain *N*‐phenylethyl groups. The base peak at *m*/*z* 80 for **56**, arising by loss of a benzyl group, indicated a nitrogen‐containing portion with three degrees of unsaturation as in pyrrole, and is also typical for its N‐alkylated derivatives. For **58** and **60** the base peak was increased by 14 Da (*m*/*z* 94) and 28 Da (*m*/*z* 108), respectively. Methylations of the pyrrole at C2 for **58** and at C2 and C5 for **60** were considered most likely. A synthesis of all three reference compounds, of **56** by a Clauson‐Kaas reaction from **33** and 2,5‐dimethoxytetrahydrofuran (**55**),^[28]^ and of **58** and **60** in a solvent‐free Paal‐Knorr reaction from **33** and the dicarbonyl compounds **57** and **59**,^[29]^ confirmed the suggested structures in all three cases (Scheme 2). Detailed proposed fragmentation pathways for all three compounds are presented in Schemes S1–S3. *N*‐(2‐Phenylethyl)pyrrole (**56**) has been reported before from *Abelmoschus esculentes* ^[30]^ and *Saccharomyces cerevisiae*,^[31]^ whereas **58** and **60** represent new natural products.

**Figure 7:**
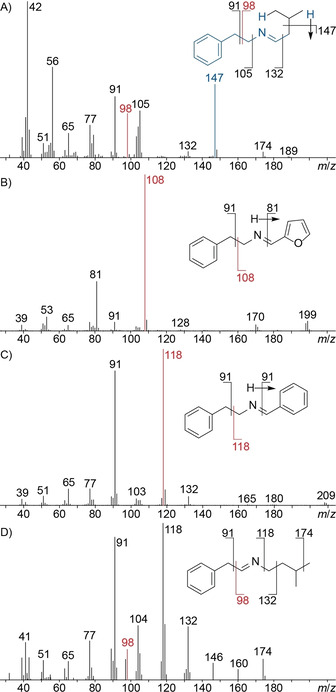
EI‐MS spectra of A) *N*‐(3‐methylbutylidene)‐2‐phenylethylamine (**62**), B) *N*‐(2‐furylmethylidene)‐2‐phenylethylamine (**64**), C) *N*‐benzylidene‐2‐phenylethylamine (**65**) and D) 3‐methyl‐*N*‐(2‐phenylethylidene)‐1‐butanamine (**67**). Red indicates fragment ions arising from benzyl group cleavage, blue indicates ions originating from McLafferty rearrangement.

**Scheme 3:**
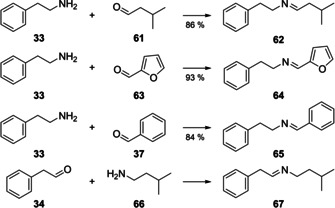
Synthesis of imine reference substances. Yields refer to condensation over molecular sieves.

Compound **62** showed a molecular ion of *m*/*z* 189 suggesting the presence of nitrogen, and fragment ions at *m*/*z* 105, 91 and 77 indicating a phenethyl group (Figure 7A). The fragment ions at *m*/*z* 56 (tentatively assigned to C_3_H_6_N^+^) and 42 (tentatively assigned to C_2_H_4_N^+^) are typical for imines, and together with the α‐fragmentation leading to *m*/*z* 132 and the neutral loss of propene through McLafferty rearrangement^[24]^ the structural suggestion of *N*‐(3‐methylbutylidene)‐2‐phenylethylamine for **62** was delineated. Another imine was tentatively identified as *N*‐(2‐furylmethylidene)‐2‐phenylethylamine (**64**) by comparison of its mass spectrum (Figure 7B) to a database spectrum. Compound **65**, one of the major constituents in the headspace (10.9 %), and **67** were identified from their mass spectra (Figure 7C and D) by comparison to library spectra as *N*‐benzylidene‐2‐phenylethylamine (**65**) and 3‐methyl‐*N*‐(2‐phenylethylidene)‐1‐butanamine (**67**), respectively. Full hypothetical fragmentation pathways for **62**, **64**, **65** and **67** are shown in Schemes S4–S7. To verify the tentatively assigned structures all four imines were prepared by condensation of the corresponding amines and aldehydes over molecular sieves (Scheme 3)^[32]^ and proved to be identical to the volatiles from *C. polytrichastri* by MS and retention index. Because of its instability compound **67** could not be isolated in pure form, but was obtained in a synthetic mixture containing one component that showed the same mass spectrum as the natural product from *C. polytrichastri*. 3‐Methyl‐*N*‐(2‐phenylethylidene)‐1‐butanamine (**67**) has been observed as a volatile from *Tuber melanosporum* before,^[33]^ whereas the imines **62**, **64** and **65** represent new natural products.

**Scheme 4:**
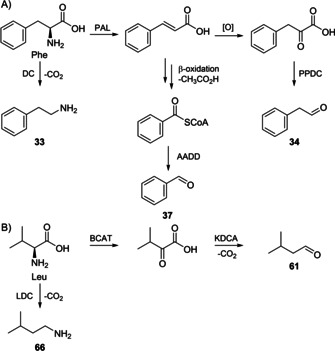
A) Compounds **33**, **34** and **37** are produced by Phe degradation, B) **61** and **66** arise from degradation of Leu. PAL: phenylalanine ammonia lyase, DC: PLP‐dependent decarboxylase, PPDC: phenyl pyruvate decarboxylase, AADD: aryl aldehyde dehydrogenase, BCAT: branched‐chain amino transferase, KDCA: α‐ketoacid decarboxylase, LDC: leucine decarboxylase.

The biosynthesis of the imines could proceed analogously to their synthesis by condensation of an aldehyde and an amine. This hypothesis is supported by the presence of several of the needed precursors in the headspace extracts, including the aldehydes **34** and **37** and the amine **33**. These building blocks can be formed by degradation of phenylalanine,[^34^, ^35^] while **61** and **66** that are not observed in the headspace can derive from leucine (Scheme 4).[^36^, ^37^] Furfural (**63**) is a sugar degradation product associated to spoilage or fermentation processes,[^38^, ^39^] but no biosynthetic pathway in bacteria was reported so far.

**Figure 8:**
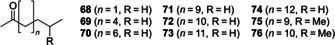
Structures of methyl ketones from *C. polytrichastri*.

One more group contributing to the headspace extract consists of methyl ketones, similar to the volatiles emitted by other *Flavobacteriaceae* (Figure 8).^[8]^ Among these are the unbranched methyl ketones **68** to **74**, spanning chain lengths from C_6_ to C_17_, and the ω−1 methyl branched compounds 12‐methyl‐2‐tridecanone (**75**) and 13‐methyl‐2‐tetradecanone (**76**), which were identified by comparison to their known mass spectra and retention indices.^[8]^

**Figure 9:**
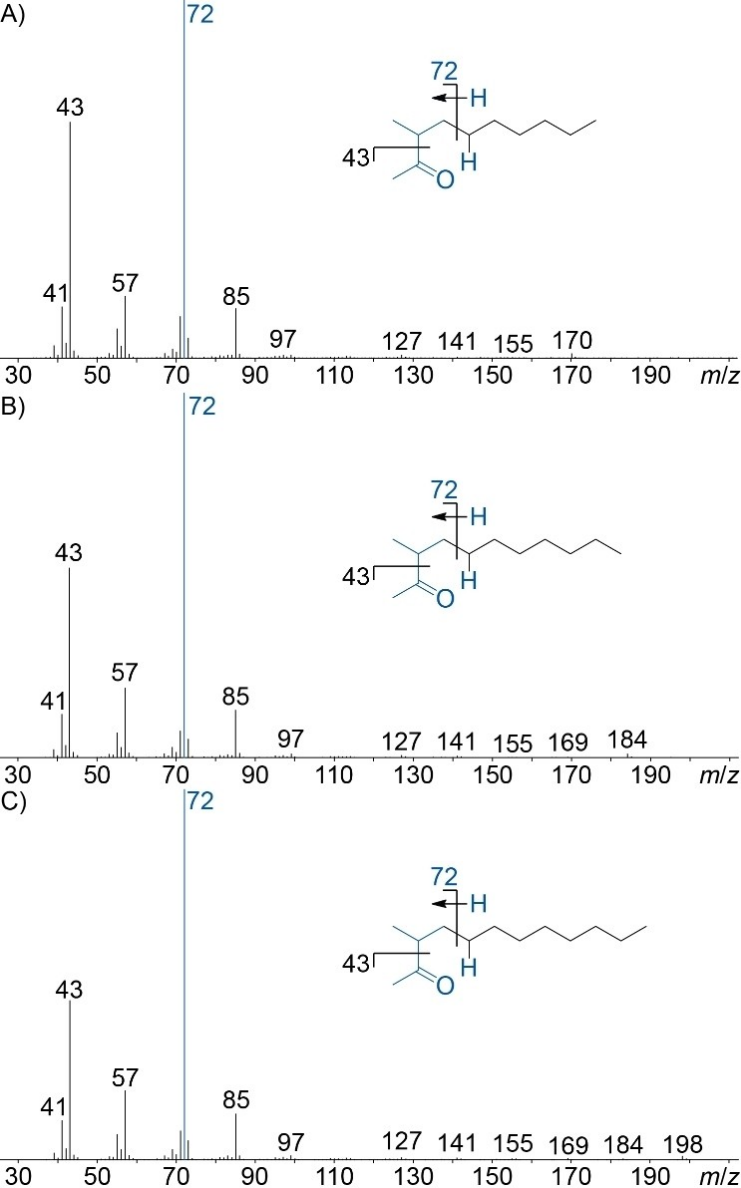
EI mass spectra of A) 3‐methyldecan‐2‐one (**84**), B) 3‐methylundecan‐2‐one (**85**) and C) 3‐methyldodecan‐2‐one (**86**). Blue indicates a McLafferty rearrangement leading to the base peak at *m*/*z* 72.

Three further compounds exhibited very similar mass spectra (Figure 9A–C), but showed molecular ions increasing by 14 Da and simultaneously increasing retention indices by 100 units, suggesting a series of structurally related homologous compounds. The observed fragment ion pattern was in agreement with 3‐methyl‐2‐ketones for which the base peak at *m*/*z* 72 is explained by McLafferty rearrangement, while the strong fragment ion at *m*/*z* 43 can arise by α‐cleavage. Furthermore, the retention indices of **85** and **86** matched recently published data for 3‐methyl‐2‐undecanone and 3‐methyl‐2‐dodecanone (Table S1).^[40]^

**Scheme 5:**
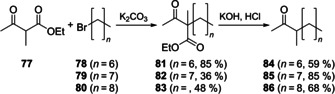
Synthesis of the methyl ketones **77**, **78** and **79**.

To confirm their identity the three ketones 3‐methyldecan‐2‐one (**84**), 3‐methylundecan‐2‐one (**85**) and 3‐methyldodecan‐2‐one (**86**) were synthesised by alkylation of ethyl 2‐methyl‐3‐oxobutanoate (**77**) with the suitable alkyl bromides (**78**–**80**), yielding the β‐keto esters **81**–**83**, which were further converted by saponification with spontaneous decarboxylation (Scheme 5). The obtained compounds showed identical mass spectra and retention indices to those of the natural products. The ketones have all been reported as natural products before, **84** in the marking fluid of *Panthera tigris*, and **85** and **86** as pheromones in *Ptomascopus morio*.[^40^, ^41^]

## 3 Conclusion

In conclusion, this study provides the first insights into the secondary metabolism of a bacterium from the genus *Chryseobacterium* by identification of the emitted volatiles. Although some similarities to distinctly related *Flavobacteriaceae* can be observed with respect to the production of methyl ketones, the most pronounced class of volatiles was represented by nitrogen‐containing compounds including pyrazines and other aromatic heterocycles, besides amides, pyrroles and imines mostly deriving from 2‐phenylethylamine. Several new natural products were identified, including *N*‐(2‐phenylethyl)pentanamide (**50**), 2‐methyl‐*N*‐(2‐phenylethyl)‐pyrrole (**58**), 2,5‐dimethyl*‐N*‐(2‐phenylethyl)pyrrole (**60**), *N*‐(3‐methylbutylidene)‐2‐phenylethylamine (**62**), *N*‐(2‐furylmethylidene)‐2‐phenylethylamine (**64**) and *N*‐benzylidene‐2‐phenylethylamine (**65**), thus demonstrating that the genus *Chryseobacterium* is of high interest to natural product chemists. This was also reflected in our recent discovery of diterpene synthases yielding diterpenes of new skeletons.^[9]^ However, the terpenes produced by the known synthases from *Chryseobacterium* were not found in the headspace extract, suggesting that the corresponding genes are not expressed under laboratory culture conditions. Some of the simple compounds reported here such as pyrazine (**9**) might (partially) originate from the medium, which is also possible for benzaldehyde (**37**) and furfural (**63**) used as building blocks in the formation of imines. Clarification of a biosynthetic origin could be obtained by feeding of isotopically labelled potential biosynthetic precursors. Future studies in our laboratories will include investigations on the volatiles released by bacteria of other untapped genera.

## Experimental Section

**General experimental details**: Reactions were performed in dried flasks under an Ar atmosphere and in dried solvents. Chemicals were used as purchased from the supplier. Cooling was maintained using ice/water (0 °C) or liquid nitrogen/acetone (−78 °C) for time periods up to 1 h. For longer periods of time a cooling unit was used. Column chromatography was performed on silica gel (0.04–0.06 nm, Acros Organics, Geel, Belgium) with previously distilled solvents.

**Analytical methods**: NMR spectroscopy was performed at 297 K either on an Avance I (400 MHz), Avance I, Avance III HD Ascend (both 500 MHz) or an Avance III HD Ascend (700 MHz) from Bruker (Billerica, MA, USA). Spectra were referenced against solvent peaks (^1^H NMR: CDCl_3_ *δ*=7.26 ppm, C_6_D_6_ *δ*=7.16 ppm; ^13^C NMR: CDCl_3_ *δ*=77.16 ppm, C_6_D_6_ *δ*=128.06 ppm).^[42]^ Multiplicities are given by d (doublet), t (triplet), q (quartet), sept (septet) and br (broad). IR spectra were recorded on a Bruker α with a diamond ATR probe head, intensities of bands are indicated by w (weak), m (medium) and s (strong). GC/MS analysis was performed on a 7890B GC–5977 A MD system (Agilent, Santa Clara, CA, USA) fitted with a HP5‐MS UI column (30 m, 0.25 mm i.d., 0.50 μm film). The GC was operated with 1) an inlet pressure of 77.1 kPa at an He flow of 23.3 mL min^−1^, 2) an injection volume of 1 μL (synthetic samples) or 2 μL (headspace extracts), 3) a temperature ramp starting with 5 min at 50 °C, increasing at 10 °C min^−1^ (synthetic) or 5 °C min^−1^ (headspace) to 320 °C, 4) 60 s valve time and 5) He carrier gas flow of 1.2 mL min^−1^. The parameters of the MS were 1) source temperature: 230 °C, 2) transfer line temperature: 250 °C, 3) quadrupole temperature: 150 °C and 4) electron energy: 70 eV. Retention indices were determined in comparison to a homologous series of *n*‐alkanes (C_7_−C_40_). GC/MS‐QTOF analyses were conducted on a 7890B GC–7200 accurate‐mass Q‐TOF detector system (Agilent). The GC was equipped with a HP5‐MS fused silica capillary column (30 m, 0.25 mm i. d., 0.50 μm film). GC parameters were 1) injection volume: 1 μL, 2) split ratio: 10 : 1, 60 s valve time, 3) carrier gas: He at 1 mL min^−1^, and 4) temperature program: 5 min at 50 °C increasing at 10 °C min^−1^ to 320 °C. MS parameters were 1) inlet pressure: 83.2 kPa, He at 24.6 mL min^−1^, 2) transfer line: 250 °C, 3) electron energy 70 eV.

**Culture conditions and preparation of headspace extracts**: *C. polytrichastri* DSM 26899 was obtained from the DSMZ (Braunschweig, Germany) and was cultivated on 123 TGY (5.0 g L^−1^ tryptone, 5.0 g L^−1^ yeast extract, 1.0 g L^−1^ glucose, 1.0 g L^−1^ K_2_HPO_4_, pH 6.9, autoclaved at 121 °C for 20 min; for solid medium 20.0 g L^−1^ agar was added before autoclaving). Liquid cultures were inoculated from glycerol stocks and were cultivated at 28 °C with shaking at 160 rpm. Plates were inoculated using 400–600 μL of a liquid culture grown for 3 days and were cultivated for 3 days at 28 °C before submitting them to the CLSA. The volatiles were collected for 24 h and were extracted from the activated charcoal using small amounts of dichloromethane (7×10 μL). The extracts were directly injected to the GC/MS.

**Synthesis of phenethyl benzoate (32)**:^[43]^ To a cooled (0 °C) solution of 2‐phenylethanol (2.10 g, 17.3 mmol, 1.0 equiv) and pyridine (1.67 mL, 20.7 mmol, 1.2 equiv) in dichloromethane (50 mL) was slowly added benzoyl chloride (2.40 mL, 20.7 mmol, 1.2 equiv) and the mixure was stirred for 18 h at room temperature. The mixture was cooled to 0 °C and the reaction was terminated by addition of water. The organic phase was washed subsequently with NaOH (1 m, 2×50 mL), HCl (1 m, 50 mL) and brine (50 mL), dried with MgSO_4_ and evaporated to dryness. The crude mixture was purified by flash chromatography (cyclohexane/ethyl acetate 5 : 1) to yield the title compound as colourless oil (2.80 g, 12.3 mmol, 72 %). TLC (cyclohexane/ethyl acetate 5 : 1): *R*_f_=0.45. ^1^H NMR (500 MHz, CDCl_3_, 298 K): *δ*=8.08 (dd, 2H, ^3^ *J*_H,H_=7.7 Hz, ^4^ *J*_H,H_=1.6 Hz), 7.57–7.62 (m, 1H), 7.48 (dd, 2H, ^3^ *J*_H,H_=6.0 Hz, 7.7 Hz), 7.32–7.40 (m, 4H), 7.26–7.32 (m, 1H), 4.59 (t, 2H, ^3^ *J*_H,H_=7.1 Hz), 3.13 (t, 2H, ^3^ *J*_H,H_=7.0 Hz) ppm. ^13^C NMR (126 MHz, CDCl_3_, 298 K): *δ*=166.6 (C_q_), 138.0 (C_q_), 133.0 (CH), 130.4 (C_q_), 129.7 (2xCH), 129.1 (2xCH), 128.7 (2xCH), 128.5 (2xCH), 126.7 (CH), 65.6 (CH_2_), 35.4 (CH_2_) ppm.

**Synthesis of** ***N*** **‐(2‐phenylethyl)formamide (46)**:^[22]^ A mixture of 2‐phenylethylamine (2.26 mL, 18.7 mmol, 1.0 equiv) and ethyl formate (30 mL, 27.5 mmol, 1.5 equiv) was stirred under reflux overnight. Excess of ethyl formate was removed under high vacuum to yield the pure amide as viscous oil (2.57 g, 17.2 mmol, 92 %; mixture of *E* and *Z* diastereomers). ^1^H NMR (500 MHz, CDCl_3_, 298 K): *δ*=8.13 (s, 1H, *Z*), 7.89 (d, 1H, ^3^ *J*_H,H_=10.9 Hz, *E*), 7.28–7.34 (m, 2H), 7.15–7.26 (m, 3H), 5.83 (br s, 1 H), 3.57 (t, 2H, ^3^ *J*_H,H_=6.4 Hz, *Z*), 3.47 (t, 1 H, ^3^ *J*_H,H_=7.0 Hz, *E*), 2.85 (t, 2H, ^3^ *J*_H,H_=6.5 Hz) ppm. ^13^C NMR (126 MHz, CDCl_3_, 298 K): *δ*=164.6 (CH, *E*), 161.4 (CH, *Z*), 138.5 (C_q_, *Z*), 129.0 (2xCH, *E*), 128.9 (2xCH, *Z*), 128.8 (4xCH, 2x*E*, 2x*Z*), 127.1 (CH, *E*), 126.8 (CH, *Z*), 43.3 (CH_2_, *E*), 39.4 (CH_2_, *Z*), 37.8 (CH_2_, *E*), 35.6 (CH_2_, *Z*) ppm.

**General procedure for the synthesis of** ***N*** **‐(2‐phenylethyl)amides**:^[25]^ To a solution of 2‐phenylethylamine (1.65 equiv) in dichloromethane (0.25 m) was added a solution of acid chloride (1.0 equiv) in dichloromethane (0.4 m). The mixture was stirred for 1 h at room temperature and was washed subsequently with NaHCO_3_ solution (5 %), HCl (1 m) and NaCl solution (1 m). The organic phase was dried with MgSO_4_ and evaporated to dryness to yield the amides in >95 % purity.

***N*** **‐(2‐Phenylethyl)acetamide (47)**: Yield: 0.216 g (1.32 mmol, 65 %). ^1^H NMR (500 MHz, CDCl_3_, 298 K): *δ*=7.30 (dd, 2H, ^3^ *J*_H,H_=6.5 Hz, 7.3 Hz), 7.22 (tt, 1H, ^3^ *J*_H,H_=7.5 Hz, ^4^ *J*_H,H_=1.5 Hz), 7.19 (d, 2H, ^3^ *J*_H,H_=6.9 Hz), 5.79 (br s, 1H), 3.51 (dt, 2H, ^3^ *J*_H,H_=6.8 Hz, 7.0 Hz), 2.82 (t, 2H, ^3^ *J*_H,H_=7.0 Hz), 1.95 (s, 3H) ppm. ^13^C NMR (126 MHz, CDCl_3_, 298 K): *δ*=170.4 (C_q_), 138.9 (C_q_), 128.9 (2xCH), 128.8 (2xCH), 126.7 (CH), 40.9 (CH_2_), 35.7 (CH_2_), 23.3 (CH_3_) ppm.

***N*** **‐(2‐Phenylethyl)propanamide (48)**: Yield: 0.326 g (1.83 mmol, 91 %). ^1^H NMR (500 MHz, CDCl_3_, 298 K): *δ*=7.30 (ddd, 2H, ^3^ *J*_H,H_=7.2 Hz, 7.3 Hz, ^5^ *J*_H,H_=1.5 Hz), 7.23 (tt, 1H, ^3^ *J*_H,H_=7.2 Hz, ^4^ *J*_H,H_=2.1 Hz), 7.19 (dd, 2H, ^3^ *J*_H,H_=7.2 Hz, ^4^ *J*_H,H_=1.5 Hz), 5.68 (br s, 1H), 3.52 (dt, 2H, ^3^ *J*_H,H_=6.4 Hz, 7.0 Hz), 2.82 (t, 2H, ^3^ *J*_H,H_=7.0 Hz), 2.17 (q, 2H, ^3^ *J*_H,H_=7.4 Hz), 1.12 (t, 3H, ^3^ *J*_H,H_=7.5 Hz) ppm. ^13^C NMR (126 MHz, CDCl_3_, 298 K): *δ*=174.1 (C_q_), 139.0 (C_q_), 128.9 (2xCH), 128.8 (2xCH), 126.6 (CH), 40.7 (CH_2_), 35.8 (CH_2_), 29.8 (CH_2_), 10.0 (CH_3_) ppm.

***N*** **‐(2‐Phenylethyl)butanamide (49)**: Yield: 0.386 g (2.0 mmol, 100 %). ^1^H NMR (500 MHz, CDCl_3_, 298 K): *δ*=7.30 (ddd, 2H, ^3^ *J*_H,H_=7.4 Hz, 7.8 Hz, ^5^ *J*_H,H_=1.4 Hz), 7.23 (tt, 1H, ^3^ *J*_H,H_=7.6 Hz, ^4^ *J*_H,H_=1.3 Hz), 7.19 (dd, 2H, ^3^ *J*_H,H_=7.8 Hz, ^4^ *J*_H,H_=1.4 Hz), 5.59 (br s, 1H), 3.52 (dt, 2H, ^3^ *J*_H,H_=5.5 Hz, 7.2 Hz), 2.82 (t, 2H, ^3^ *J*_H,H_=7.0 Hz), 2.11 (t, 2H, ^3^ *J*_H,H_=7.4 Hz), 1.62 (tq, 2H, ^3^ *J*_H,H_=7.4 Hz, 7.2 Hz), 0.91 (t, 3H, ^3^ *J*_H,H_=7.2 Hz) ppm. ^13^C NMR (126 MHz, CDCl_3_, 298 K): *δ*=173.2 (C_q_), 139.0 (C_q_), 128.9 (2xCH), 128.7 (2xCH), 126.6 (CH), 40.7 (CH_2_), 38.8 (CH_2_), 35.8 (CH_2_), 19.3 (CH_2_), 13.9 (CH_3_) ppm.

***N*** **‐(2‐Phenylethyl)pentanamide (50)**: Yield: 0.325 g (1.58 mmol, 78 %). ^1^H NMR (500 MHz, CDCl_3_, 298 K): *δ*=7.31 (ddd, 2H, ^3^ *J*_H,H_=7.3 Hz, 6.8 Hz, ^5^ *J*_H,H_=1.5 Hz), 7.23 (tt, 1H, ^3^ *J*_H,H_=7.4 Hz, ^4^ *J*_H,H_=1.4 Hz), 7.19 (dd, 2H, ^3^ *J*_H,H_=7.0 Hz, ^4^ *J*_H,H_=1.4 Hz), 5.68 (br s, 1H), 3.52 (dt, 2H, ^3^ *J*_H,H_=5.9 Hz, 6.9 Hz), 2.81 (t, 2H, ^3^ *J*_H,H_=7.0 Hz), 2.14 (t, 2H, ^3^ *J*_H,H_=7.6 Hz), 1.57 (tt, 2H, ^3^ *J*_H,H_=7.4 Hz, 7.5 Hz), 1.31 (tq, 2H, ^3^ *J*_H,H_=7.5 Hz, 7.6 Hz), 0.91 (t, 3H, ^3^ *J*_H,H_=7.4 Hz) ppm. ^13^C NMR (126 MHz, CDCl_3_, 298 K): *δ*=173.4 (C_q_), 139.0 (C_q_), 128.9 (2xCH), 128.8 (2xCH), 126.6 (CH), 40.7 (CH_2_), 36.6 (CH_2_), 35.8 (CH_2_), 28.0 (CH_2_), 22.5 (CH_2_), 13.9 (CH_3_) ppm.

***N*** **‐(2‐Phenylethyl)hexanamide (51)**: Yield: 0.386 g (1.76 mmol, 87 %). ^1^H NMR (500 MHz, CDCl_3_, 298 K): *δ*=7.31 (dd, 2H, ^3^ *J*_H,H_=7.4 Hz, 7.6 Hz), 7.23 (t, 1H, ^3^ *J*_H,H_=7.5 Hz), 7.19 (dd, 2H, ^3^ *J*_H,H_=7.4 Hz, ^4^ *J*_H,H_=1.2 Hz), 5.46 (br s, 1H), 3.52 (dt, 2H, ^3^ *J*_H,H_=6.1 Hz, 6.8 Hz), 2.81 (t, 2H, ^3^ *J*_H,H_=7.0 Hz), 2.11 (t, 2H, ^3^ *J*_H,H_=7.7 Hz), 1.58 (tt, 2H, ^3^ *J*_H,H_=7.2 Hz, 7.7 Hz), 1.22–1.35 (m, 4H), 0.88 (t, 3H, ^3^ *J*_H,H_=7.0 Hz) ppm. ^13^C NMR (126 MHz, CDCl_3_, 298 K): *δ*=173.2 (C_q_), 139.1 (C_q_), 128.9 (2xCH), 128.7 (2xCH), 126.6 (CH), 40.6 (CH_2_), 36.9 (CH_2_), 35.9 (CH_2_), 31.5 (CH_2_), 25.5 (CH_2_), 22.5 (CH_2_), 14.0 (CH_3_) ppm.

***N*** **‐(2‐Phenylethyl)octanamide (52)**: Yield: 0.443 g (1.80 mmol, 89 %). ^1^H NMR (500 MHz, CDCl_3_, 298 K): *δ*=7.31 (ddd, 2H, ^3^ *J*_H,H_=7.4 Hz, 7.4 Hz, ^5^ *J*_H,H_=1.5 Hz), 7.23 (tt, 1H, ^3^ *J*_H,H_=7.4 Hz, ^4^ *J*_H,H_=1.4 Hz), 7.19 (dd, 2H, ^3^ *J*_H,H_=7.4 Hz, ^4^ *J*_H,H_=1.3 Hz), 5.63 (br s, 1H), 3.52 (dt, 2H, ^3^ *J*_H,H_=6.2 Hz, 6.9 Hz), 2.82 (t, 2H, ^3^ *J*_H,H_=7.0 Hz), 2.13 (t, 2H, ^3^ *J*_H,H_=7.4 Hz), 1.58 (tt, 2H, ^3^ *J*_H,H_=7.2 Hz, 7.4 Hz), 1.22–1.31 (m, 8H), 0.87 (t, 3H, ^3^ *J*_H,H_=6.9 Hz) ppm. ^13^C NMR (126 MHz, CDCl_3_, 298 K): *δ*=173.5 (C_q_), 139.0 (C_q_), 128.9 (2xCH), 128.8 (2xCH), 126.7 (CH), 40.7 (CH_2_), 36.9 (CH_2_), 35.8 (CH_2_), 31.8 (CH_2_), 29.4 (CH_2_), 29.1 (CH_2_), 25.9 (CH_2_), 22.7 (CH_2_), 14.2 (CH_3_) ppm.

***N*** **‐(2‐Phenylethyl)‐3‐methylbutanamide (53)**: Yield: 0.378 g (1.84 mmol, 92 %). ^1^H NMR (500 MHz, CDCl_3_, 298 K): *δ*=7.31 (dd, 2H, ^3^ *J*_H,H_=7.2 Hz, 7.4 Hz), 7.22 (t, 1H, ^3^ *J*_H,H_=7.2 Hz), 7.19 (d, 2H, ^3^ *J*_H,H_=7.2 Hz), 5.83 (br s, 1H), 3.54 (dt, 2H, ^3^ *J*_H,H_=6.2 Hz, 6.6 Hz), 2.83 (t, 2H, ^3^ *J*_H,H_=7.0 Hz), 2.08 (tsept, 1H, ^3^ *J*_H,H_=7.0 Hz, 6.4 Hz), 2.02 (d, 2H, ^3^ *J*_H,H_=7.0 Hz), 0.91 (d, 6H, ^3^ *J*_H,H_=6.4 Hz) ppm. ^13^C NMR (126 MHz, CDCl_3_, 298 K): *δ*=173.0 (C_q_), 139.0 (C_q_), 128.9 (2xCH), 128.8 (2xCH), 126.7 (CH), 46.0 (CH_2_), 40.8 (CH_2_), 35.8 (CH_2_), 26.3 (CH), 22.5 (2xCH_3_) ppm.

***N*** **‐(2‐Phenylethyl)benzamide (54)**: Yield: 0.800 g (1.32 mmol, 65 %). ^1^H NMR (500 MHz, CDCl_3_, 298 K): *δ*=7.70 (ddd, 2H, ^3^ *J*_H,H_=8.4 Hz, ^4^ *J*_H,H_=1.5 Hz, ^5^ *J*_H,H_=3.1 Hz), 7.48 (tt, 1 H, ^3^ *J*_H,H_=6.5 Hz, ^4^ *J*_H,H_=1.4 Hz), 7.40 (ddd, 2H, ^3^ *J*_H,H_=8.3 Hz, 7.7 Hz, ^4^ *J*_H,H_=1.5 Hz), 7.33 (ddd, 2H, ^3^ *J*_H,H_=6.5 Hz, 6.5 Hz, ^4^ *J*_H,H_=1.5 Hz), 7.23–7.27 (m, 3H), 6.20 (br s, 1H), 3.72 (dt, 2H, ^3^ *J*_H,H_=5.9 Hz, 6.9 Hz), 2.94 (t, 2H, ^3^ *J*_H,H_=6.9 Hz) ppm. ^13^C NMR (126 MHz, CDCl_3_, 298 K): *δ*=167.6 (C_q_), 139.0 (C_q_), 134.8 (C_q_), 131.5 (CH), 128.9 (2xCH), 128.8 (2xCH), 128.7 (2xCH), 126.9 (2xCH), 126.7 (CH), 41.3 (CH_2_), 35.8 (CH_2_) ppm.

**Clauson‐Kaas reaction to 56**:^[29]^ To a solution of 2,5‐dimethoxytetrahydrofuran (3.93 mL, 29.2 mmol, 1.0 equiv) in glacial acetic acid (15 mL) was added 2‐phenylethylamine (3.68 mL, 29.2 mmol, 1.0 equiv). After stirring the reaction mixture under reflux for 1 h, acetic acid was removed at the rotary evaporator. The residue was redissolved in ethyl acetate (150 mL). The organic layer was washed subsequently with HCl (1 m), K_2_CO_3_ solution (10 %) and brine (150 mL each), dried with MgSO_4_ and evaporated to dryness. The residue was subjected to column chromatography (cyclohexane/ethyl acetate 15 : 1) to yield **56** as colourless oil (3.81 g, 22.2 mmol, 66 %). TLC (cyclohexane/ethyl acetate 20 : 1) *R*_f_=0.46. ^1^H NMR (500 MHz, CDCl_3_, 298 K): *δ*=7.30 (dd, 2H, ^3^ *J*_H,H_=6.5 Hz, 7.4 Hz), 7.11 (d, 2H, ^3^ *J*_H,H_=7.3 Hz), 7.22–7.27 (m, 1H), 6.61 (s, 2H), 6.14 (s, 2H), 4.12 (t, 2H, ^3^ *J*_H,H_=7.5 Hz), 3.06 (t, 2H, ^3^ *J*_H,H_=7.6 Hz) ppm. ^13^C NMR (126 MHz, CDCl_3_, 298 K): *δ*=138.6 (C_q_), 128.8 (2xCH), 128.7 (2xCH), 126.7 (CH), 120.6 (2xCH), 108.1 (2xCH), 51.3 (CH_2_), 38.5 (CH_2_) ppm.

**General procedure for Paal‐Knorr synthesis**:^[30]^ A mixture of 2‐phenylethylamine (1.0 equiv) and a 1,4‐dicarbonyl compound (**57**, **59**; 1.0 equiv) was stirred at room temperature for 1 h. The water formed during the reaction was removed under high vacuum. The products were purified by column chromatography (cyclohexane/ethyl acetate 30 : 1).

**2‐Methyl‐*N*‐(2‐phenylethyl)pyrrole (58)**: Yield: 0.880 g (4.75 mmol, 95 %). TLC (cyclohexane/ethyl acetate 30 : 1) *R*_f_=0.28. ^1^H NMR (500 MHz, CDCl_3_, 298 K): *δ*=7.33 (ddd, 2H, ^3^ *J*_H,H_=7.1 Hz, 8.1 Hz, ^5^ *J*_H,H_=1.3 Hz), 7.27 (tt, 1H, ^3^ *J*_H,H_=8.1 Hz, ^4^ *J*_H,H_=2.1 Hz), 7.13 (dd, 2H, ^3^ *J*_H,H_=7.2 Hz, ^5^ *J*_H,H_=1.3 Hz), 6.56 (br s, 1H), 6.08 (s, 1H), 5.89 (br s, 1H), 4.04 (t, 2H, ^3^ *J*_H,H_=7.5 Hz), 3.02 (t, 2H, ^3^ *J*_H,H_=7.5 Hz), 2.14 (s, 3H) ppm. ^13^C NMR (126 MHz, CDCl_3_, 298 K): *δ*=138.6 (C_q_), 128.9 (2xCH), 128.7 (2xCH), 128.4 (C_q_), 126.7 (CH), 119.7 (CH), 106.9 (CH), 106.6 (CH), 48.3 (CH_2_), 38.3 (CH_2_), 11.9 (CH_3_) ppm, (Figures S3–S5). HRMS (EI): *m*/*z*: 185.1202 (calc. for [C_13_H_15_N]^+^ 185.1199). EI‐MS (70 eV): *m*/*z* (%): 185 (34), 104 (20), 94 (100), 78 (11), 65 (7), 53 (6), 51 (4), 41 (6), 39 (5; Figure S3, Scheme S1). IR (diamond ATR): $\tilde{\nu }$ =3027 (w), 2929 (w), 2861 (w), 1682 (m), 1603 (w), 1553 (w), 1493 (m), 1453 (m), 1420 (m), 1360 (w), 1295 (m), 1236 (w), 1156 (w), 1140 (w), 1074 (w), 1029 (w), 974 (w), 887 (w), 751 (m), 732 (m), 696 (s), 614 (m), 563 (m), 495 (m) cm^−1^.

**2,5‐Dimethyl‐*N*‐(2‐phenylethyl)pyrrole (60)**: Yield: 1.658 g (8.33 mmol, 83 %). TLC (cyclohexane/ethyl acetate 30 : 1) *R*_f_=0.31. ^1^H NMR (500 MHz, CDCl_3_, 298 K): *δ*=7.33 (ddd, 2H, ^3^ *J*_H,H_=7.1 Hz, 8.1 Hz, ^5^ *J*_H,H_=1.3 Hz), 7.26–7.30 (m, 1H), 5.80 (br s, 2H), 7.15 (d, 2H, ^3^ *J*_H,H_=7.5 Hz), 3.98 (t, 2H, ^3^ *J*_H,H_=7.5 Hz), 2.93 (t, 2H, ^3^ *J*_H,H_=7.5 Hz), 2.19 (s, 6H) ppm. ^13^C NMR (126 MHz, CDCl_3_, 298 K): *δ*=138.7 (C_q_), 129.0 (2xCH), 128.7 (2xCH), 127.5 (2xC_q_), 126.8 (CH), 105.3 (2xCH), 45.4 (CH_2_), 37.7 (CH_2_), 12.5 (2xCH_3_) ppm.

**General procedure for the synthesis of imines**:^[31]^ To a stirred suspension of activated molecular sieves (4 Å, 0.5 g mol^−1^) in diethyl ether (3 mL mmol^−1^) the aldehyde (**34**, **37**, **61**, **63**; 1.0 equiv) and the amine (**33**, **66**; 1.0 equiv) were added. The mixture was stirred overnight and the molecular sieves were filtered off and washed with diethyl ether. The filtrate was evaporated to dryness to yield the imines in high purity (>95 %).

***N*** **‐(3‐Methylbutylidene)‐2‐phenylethylamine (62)**: Yield: 8.14 g (43 mmol, 86 %). ^1^H NMR (500 MHz, CDCl_3_, 298 K): *δ*=7.53 (t, 1H, ^3^ *J*_H,H_=5.7 Hz), 7.26–7.33 (m, 2H), 7.16–7.22 (m, 3H), 3.65 (t, 2H, ^3^ *J*_H,H_=7.4 Hz), 2.94 (t, 2H, ^3^ *J*_H,H_=7.4 Hz), 2.08–2.15 (m, 2H), 1.85 (sept, 1H, ^3^ *J*_H,H_=6.6 Hz), 0.90 (d, 6H, ^3^ *J*_H,H_=6.6 Hz) ppm. ^13^C NMR (126 MHz, CDCl_3_, 298 K): *δ*=165.4 (CH), 140.0 (C_q_), 129.1 (2xCH), 128.4 (2xCH), 126.1 (CH), 63.0 (CH_2_), 44.8 (CH_2_), 37.5 (CH_2_), 26.4 (CH), 22.6 (2xCH_3_) ppm (Figures S6–S8). HRMS (EI): *m*/*z*: 189.1512 (calc. for [C_13_H_19_N]^+^ 189.1512). EI‐MS (70 eV) *m*/*z* (%): 189 (1), 188 (1), 174 (3), 147 (47), 132 (4), 105 (28), 104 (19), 98 (27), 91 (37), 77(24), 65 (15), 56 (60), 42 (100), 39 (13; Figure 7, Scheme S4). IR (diamond ATR): $\tilde{\nu }$ =3062 (w), 3027 (w), 2953 (s), 2867 (m), 1653 (m), 1603 (w), 1495 (w), 1454 (m), 1363 (m), 1254 (w), 1166 (m), 1089 (w), 1030 (w), 1002 (w), 922 (w), 868 (w), 744 (s), 697 (s), 571 (w), 497 (m) cm^−1^.

***N*** **‐(2‐Furylmethylidene)‐2‐phenylethylamine (64)**: Yield: 9.27 g (46.5 mmol, 93 %). ^1^H NMR (500 MHz, C_6_D_6_, 298 K): *δ*=7.71 (brs, 1H), 5.97–5.99 (m, 1H), 7.12 (dd, 2H, ^3^ *J*_H,H_=7.2 Hz, 7.5 Hz), 7.09 (d, 2H, ^3^ *J*_H,H_=7.4 Hz), 7.04 (tt, 1H, ^3^ *J*_H,H_=7.2 Hz, ^4^ *J*_H,H_=1.6 Hz), 7.00 (brs, 1H), 6.59 (d, 1H, ^3^ *J*_H,H_=3.3 Hz), 3.61 (td, 2H, ^3^ *J*_H,H_=7.3 Hz, ^4^ *J*_H,H_=1.2 Hz), 2.90 (t, 2H, ^3^ *J*_H,H_=7.3 Hz) ppm. ^13^C NMR (126 MHz, C_6_D_6_, 298 K): *δ*=153.0 (C_q_), 150.0 (CH), 144.2 (CH), 140.4 (C_q_), 129.4 (2xCH), 128.6 (2xCH), 126.4 (1 C,CH), 112.1 (CH), 111.7 (CH), 63.6 (CH_2_), 37.9 (CH_2_) ppm.

***N*** **‐Benzylidene‐2‐phenylethylamine (65)**: Yield: 8.81 g (42.1 mmol, 84 %). ^1^H NMR (400 MHz, C_6_D_6_, 298 K): *δ*=7.88 (t, 1H, ^4^ *J*_H,H_=1.4 Hz), 7.71 (dd, 2H, ^3^ *J*_H,H_=7.8 Hz, ^4^ *J*_H,H_=1.6 Hz), 7.07–7.21 (m, 8H), 3.74 (td, 2H, ^3^ *J*_H,H_=7.3 Hz, ^4^ *J*_H,H_=1.3 Hz), 2.98 (t, 2H, ^3^ *J*_H,H_=7.3 Hz) ppm. ^13^C NMR (100 MHz, C_6_D_6_, 298 K): *δ*=160.8 (CH), 140.6 (C_q_), 137.1 (C_q_), 130.5 (CH), 129.4 (2xCH), 128.7 (2xCH), 128.6 (2xCH), 128.5 (2xCH), 126.4 (CH), 63.4 (CH_2_), 38.0 (CH_2_) ppm.

**3‐Methyl‐*N*‐(2‐phenylethylidene)butanamine (67)**: EI‐MS (70 eV) *m*/*z* (%): 175 (7), 174 (20), 160 (9), 146 (11), 132 (33), 118 (100), 104 (24), 98 (23), 91 (55), 77 (9), 65 (4), 55 (3), 43 (6), 41 (5; Figure 7, Scheme S7).

**General procedure for the synthesis of ethyl 2‐acetyl‐2‐methylalkanoates (81–83)**: To a suspension of potassium carbonate (2.0 equiv) in acetone (0.25 m), ethyl 2‐methylacetoacetate (3.0 equiv) and alkyl bromide (**78**–**80**; 1.0 equiv) were added. The reaction mixture was refluxed overnight and filtered after cooling to room temperature. The filter was washed with acetone and the filtrate was dried with MgSO_4_ and evaporated to dryness. The residue was subjected to column chromatography (cyclohexane/ethyl acetate 10 : 1) to yield the pure ethyl 2‐acetyl‐2‐methylalkanoates (**81**–**83**) as colourless oils.

**Ethyl 2‐acetyl‐2‐methylnonanoate (81)**: Yield: 0.377 g (1.65 mmol, 33 %). TLC (cyclohexane/ethyl acetate 10 : 1) *R*_f_=0.33. ^1^H NMR (700 MHz, CDCl_3_, 298 K): *δ*=4.18 (q, 2H, ^3^ *J*_H,H_=7.1 Hz), 2.13 (s, 3H), 1.83–1.90 (m, 1H), 1.69–1.77 (m, 1H), 1.31 (s, 3H), 1.20 −1.30 (m, 8H), 1.25 (t, 3H, ^3^ *J*_H,H_=7.1 Hz), 1.11–1.17 (m, 2H), 0.87 (t, 3H, ^3^ *J*_H,H_=7.1 Hz) ppm. ^13^C NMR (176 MHz, CDCl_3_, 298 K): *δ*=206.0 (C_q_), 173.3 (C_q_), 61.3 (CH_2_), 59.9 (C_q_), 34.9 (CH_2_), 31.9 (CH_2_), 30.1 (CH_2_), 29.2 (CH_2_), 26.3 (CH_3_), 24.3 (CH_2_), 22.7 (CH_2_), 18.9 (CH_3_), 14.2 (2xCH_3_) ppm (Figures S9–S11). HRMS (EI): *m*/*z*: 200.1775 (calculated for the McLaffety fragment ion [C_14_H_26_O_3_–C_2_H_2_O]^+^ 200.1771, the molecular ion was not observed). EI‐MS (70 eV) *m*/*z* (%): 200 (48), 171 (12), 157 (15), 144 (36), 129 (7), 115 (100), 98 (18), 87 (57), 69 (16), 55 (15), 43 (62), 41 (19; Figure S12). IR (diamond ATR): $\tilde{\nu }$ =2955 (m), 2927 (m), 2857 (w), 1739 (m), 1712 (s), 1463 (w), 1377 (w), 1356 (w), 1298 (w), 1240 (s), 1179 (m), 1144 (s), 1123 (m), 1096 (m), 1024 (m), 970 (w), 860 (w), 807 (w), 769 (w), 723 (w), 602 (w), 539 (w) cm^−1^.

**Ethyl 2‐acetyl‐2‐methyldecanoate (82)**: Yield: 0.464 g (1.81 mmol, 36 %). TLC (cyclohexane/ethyl acetate 10 : 1) *R*_f_=0.36. ^1^H NMR (500 MHz, CDCl_3_, 298 K): *δ*=4.18 (q, 2H, ^3^ *J*_H,H_=7.2 Hz), 2.13 (s, 3H), 1.31 (s, 3H), 1.83–1.90 (m, 1H), 1.69–1.77 (m, 1H), 1.22–1.30 (m, 10H), 1.25 (t, 3H, ^3^ *J*_H,H_=7.2 Hz), 1.10–1.18 (m, 2H), 0.87 (t, 3H, ^3^ *J*_H,H_=7.2 Hz) ppm. ^13^C NMR (126 MHz, CDCl_3_, 298 K): *δ* =206.0 (C_q_), 173.3 (C_q_), 61.3 (CH_2_), 59.8 (C_q_), 34.9 (CH_2_), 32.0 (CH_2_), 30.1 (CH_2_), 29.5 (CH_2_), 29.3 (CH_2_), 26.3 (CH_3_), 24.3 (CH_2_), 22.8 (CH_2_), 18.9 (CH_3_), 14.2 (2xCH_3_) ppm (Figures S13–S15). HRMS (EI): *m*/*z*: 214.1928 (calculated for the McLaffety fragment ion [C_15_H_28_O_3_ −C_2_H_2_O]^+^ 214.1927, the molecular ion was not observed). EI‐MS (70 eV) *m*/*z* (%): 214 (35), 171 (15), 157 (20), 144 (36), 129 (7), 115 (100), 98 (19), 87 (60), 69 (20), 55 (21), 43 (84), 41 (32; Figure S12). IR (diamond ATR): $\tilde{\nu }$ =2924 (s), 2854 (m), 1740 (m), 1712 (s), 1463 (w), 1377 (w), 1356 (w), 1243 (s), 1229 (m), 1175 (m), 1144 (s), 1125 (m), 1099 (m), 1022 (m), 971 (w), 859 (w), 770 (w), 722 (w), 603 (w), 540 (w) cm^−1^.

**Ethyl 2‐acetyl‐2‐methylundecanoate (83)**: Yield: 0.648 g (2.40 mmol, 48 %). TLC (cyclohexane/ethyl acetate 10 : 1) *R*_f_=0.41. ^1^H NMR (500 MHz, CDCl_3_, 298 K): *δ*=4.18 (q, 2H, ^3^ *J*_H,H_=7.1 Hz), 2.13 (s, 3H), 1.83–1.90 (m, 1H), 1.69–1.77 (m, 1H), 1.31 (s, 3H), 1.22–1.30 (m, 12H), 1.26 (t, 3H, ^3^ *J*_H,H_=7.2 Hz), 1.11–1.19 (m, 2H), 0.87 (t, 3H, ^3^ *J*_H,H_=7.0 Hz) ppm. ^13^C NMR (176 MHz, CDCl_3_, 298 K): *δ*=206.0 (C_q_), 173.3 (C_q_), 61.3 (CH_2_), 59.9 (C_q_), 34.9 (CH_2_), 32.0 (CH_2_), 30.1 (CH_2_), 29.6 (CH_2_), 29.5 (CH_2_), 29.4 (CH_2_), 26.3 (CH_3_), 24.3 (CH_2_), 22.8 (CH_2_), 18.9 (CH_3_), 14.2 (2xCH_3_) ppm (Figures S16–S18). HRMS (EI): *m*/*z*: 228.2083 (calculated for the McLaffety fragment ion [C_16_H_30_O_3_ −C_2_H_2_O]^+^ 228.2084, the molecular ion was not observed). EI‐MS (70 eV) *m*/*z* (%): 228 (93), 199 (3), 171 (36), 157 (14), 144 (47), 129 (8), 115 (100), 98 (16), 87 (48), 69 (17), 55 (18), 43 (93), 41 (30; Figure S12). IR (diamond ATR): $\tilde{\nu }$ =2925 (s), 2855 (w), 1740 (m), 1713 (s), 1621 (w), 1463 (w), 1377 (w), 1356 (w), 1234 (s), 1176 (m), 1144 (s), 1124 (m), 1099 (s), 1022 (m), 971 (w), 859 (w), 808 (w), 768 (w), 723 (w), 603 (w), 539 (w) cm^−1^.

**General procedure for the synthesis of 3‐methylalkan‐2‐ones (84–86)**: To a solution of the ethyl 2‐acetyl‐2‐methylalkanoates (**81**–**83**; 1.0 equiv) in ethanol (0.2 m) was added potassium hydroxide solution (3 m, 2.0 equiv) and the mixture was refluxed for 3 h. After cooling to room temperature HCl (3 m) was added and the mixture was diluted with ethyl acetate The separated organic phase was washed with water, dried with MgSO_4_ and evaporated to dryness. The residue was purified by column chromatography (cyclohexane/ethyl acetate 20 : 1) to give the 3‐methylalkan‐2‐ones (**84**–**86**) as colourless oils.

**3‐Methyl‐2‐decanone (84)**: Yield: 0.166 g (0.98 mmol, 59 %). TLC (cyclohexane/ethyl acetate 20 : 1) *R*_f_=0.28. ^1^H NMR (700 MHz, CDCl_3_, 298 K): *δ*=2.49 (ddq, 1H, ^3^ *J*_H,H_=6.9 Hz, 6.9 Hz, 6.9 Hz), 2.12 (s, 3H), 1.60–1.66 (m, 1H), 1.20–1.36 (m, 11H), 1.07 (d, 3H, ^3^ *J*_H,H_=6.9 Hz), 0.87 (t, 3H, ^3^ *J*_H,H_=6.9 Hz) ppm. ^13^C NMR (176 MHz, CDCl_3_, 298 K): *δ*=213.1 (C_q_), 47.4 (CH), 33.1 (CH_2_), 31.9 (CH_2_), 29.8 (CH_2_), 29.3 (CH_2_), 28.1 (CH_3_), 27.4 (CH_2_), 22.8 (CH_2_), 16.3 (CH_3_), 14.2 (CH_3_) ppm (Figures S19–S21). HRMS (EI): *m*/*z*: 170.1671 (calc. for [C_11_H_22_O]^+^ 170.1665). EI‐MS (70 eV) *m*/*z* (%): 170 (2), 85 (14), 72 (100), 57 (17), 55 (8), 43 (60), 41 (12; Figure 9), IR (diamond ATR): $\tilde{\nu }$ =2958 (m), 2925 (s), 2855 (m), 1712 (s), 1461 (m), 1356 (m), 1237 (w), 1168 (w), 1138 (w), 1106 (w), 953 (w), 806 (w), 722 (w), 601 (w), 496 (w) cm^−1^.

**3‐Methyl‐2‐undecanone (85)**: Yield: 0.284 g (1.54 mmol, 85 %). TLC (cyclohexane/ethyl acetate 20 : 1) *R*_f_=0.31. ^1^H NMR (500 MHz, CDCl_3_, 298 K): *δ*=2.49 (ddq, 1H, ^3^ *J*_H,H_=6.9 Hz, 6.9 Hz, 6.9 Hz), 2.12 (s, 3H), 1.58–1.67 (m, 1H), 1.20–1.37 (m, 13H), 1.06 (d, 3H, ^3^ *J*_H,H_=7.0 Hz), 0.87 (t, 3H, ^3^ *J*_H,H_=6.9 Hz) ppm. ^13^C NMR (176 MHz, CDCl_3_, 298 K): *δ*=213.2 (C_q_), 47.4 (CH), 33.1 (CH_2_), 32.0 (CH_2_), 29.8 (CH_2_), 29.6 (CH_2_), 29.4 (CH_2_), 28.1 (CH_3_), 27.4 (CH_2_), 22.8 (CH_2_), 16.3 (CH_3_), 14.2 (CH_3_) ppm (Figures S22–S24). HRMS (EI): *m*/*z*: 184.1821 (calc. for [C_12_H_24_O]^+^ 184.1822). EI‐MS (70 eV) *m*/*z* (%): 184 (1), 99 (1), 85 (13), 72 (100), 57 (19), 55 (7), 43 (52), 41 (12; Figure 9). IR (diamond ATR): $\tilde{\nu }$ =2958 (m), 2924 (s), 2855 (m), 1712 (s), 1461 (m), 1356 (m), 1232 (w), 1166 (w), 1137 (w), 1106 (w), 952 (w), 804 (w), 721 (w), 602 (w), 496 (w) cm^−1^.

**3‐Methyl‐2‐dodecanone (86)**: Yield: 0.325 g (1.64 mmol, 68 %). TLC (cyclohexane/ethyl acetate 20 : 1) *R*_f_=0.35. ^1^H NMR (500 MHz, CDCl_3_, 298 K): *δ*=2.49 (ddq, 1H, ^3^ *J*_H,H_=6.9 Hz, 6.9 Hz, 6.9 Hz), 2.12 (s, 3H), 1.59–1.67 (m, 1H), 1.20–1.37 (m, 15H), 1.07 (d, 3H, ^3^ *J*_H,H_=7.0 Hz), 0.87 (t, 3H, ^3^ *J*_H,H_=6.9 Hz) ppm. ^13^C NMR (176 MHz, CDCl_3_, 298 K): *δ*=213.2 (C_q_), 47.4 (CH), 33.1 (CH_2_), 32.0 (CH_2_), 29.8 (CH_2_), 29.7 (CH_2_), 29.6 (CH_2_), 29.4 (CH_2_), 28.1 (CH_3_), 27.4 (CH_2_), 22.8 (CH_2_), 16.3 (CH_3_), 14.2 (CH_3_) ppm (Figures S25–S27). HRMS (EI): *m*/*z*: 198.1981 (calc. for [C_13_H_26_O]^+^ 198.1978). EI‐MS (70 eV) *m*/*z* (%): 198 (1), 99 (1), 85 (14), 72 (100), 57 (22), 55 (8), 43 (52), 41 (13; Figure 9). IR (diamond ATR): $\tilde{\nu }$ =2958 (m), 2924 (s), 2854 (m), 1712 (s), 1461 (m), 1356 (m), 1230 (w), 1165 (w), 1137 (w), 1107 (w), 952 (w), 720 (w), 602 (w), 496 (w) cm^−1^.

## Conflict of interest

The authors declare no conflict of interest.

## Supporting information

As a service to our authors and readers, this journal provides supporting information supplied by the authors. Such materials are peer reviewed and may be re‐organized for online delivery, but are not copy‐edited or typeset. Technical support issues arising from supporting information (other than missing files) should be addressed to the authors.

Supplementary
