# Supplementary material for: Volatiles from the Psychrotolerant Bacterium Chryseobacterium polytrichastri
Source: Chembiochem. 2020 Sep 16;21(24):3608–17. doi: 10.1002/cbic.202000503 (PMC7756357; doi:10.1002/cbic.202000503)
Supplement: Supplementary file 1 — Supplementary [file CBIC-21-3608-s001.pdf]

ChemBioChem

Supporting Information

Volatiles from the Psychrotolerant Bacterium *Chryseobacterium polytrichastri*

Lukas Lauterbach and Jeroen S. Dickschat*

Table S1. Compounds identified in headspace extracts obtained from CLSA analyses of agar plates with *C. polytrichastri*.[a]

**compound**	**t_R / min**	***I***	***I*[lit.]**	**area**	**Ident.**
pyrazine (**9**)	3.82	731	734[1]	<0.1%	MS, *I*
dimethyl disulfide (**6**)	4.11	743	734[2]	0.1%	MS, *I*
2,3-butanediol (**25**)	5.05	779	785[3]	<0.1%	MS, *I*
2-hexanone (**68**)	5.48	795	788[2]	<0.1%	MS, *I*
methylpyrazine (**10**)	6.54	825	821[4]	0.7%	MS, *I*
2,5-dimethylpyrazine (**11**)	9.80	911	912[4]	9.2%	MS, *I*
benzaldehyde (**37**)	11.71	963	952[3]	1.0%	MS, *I*
dimethyl trisulfide (**7**)	12.06	972	983[5]	0.1%	MS, *I*
benzonitrile (**38**)	12.61	987	973[2]	<0.1%	MS, *I*
6-methylhept-5-en-2-one (**1**)	12.70	989	981[3]	<0.1%	MS, *I*
2,4,6-trimethylpyridine (**23**)	12.78	991	996[6]	0.1%	com, *I*
methanesulfonamide (**8**)	12.98	997		0.1%	MS
2-ethyl-5-methylpyrazine (**12**)	13.12	1000	997[7]	0.9%	MS, *I*
trimethylpyrazine (**13**)	13.18	1002	1002[4]	0.9%	MS, *I*
2-acetylthiazole (**21**)	13.79	1020	1014[3]	0.3%	MS, *I*
benzyl alcohol (**35**)	14.32	1035	1026[3]	0.1%	MS, *I*
phenylacetaldehyde (**34**)	14.71	1047	1036[3]	<0.1%	MS, *I*
2-isopropyl-5-methylpyrazine (**14**)	14.98	1055	1067[4]	0.1%	MS, *I*
2-acetylpyrrole (**22**)	15.24	1062	1054[3]	0.1%	MS, *I*
2-cyanopyridine (**24**)	15.39	1067	1069[8]	<0.1%	MS, *I*
acetophenone (**40**)	15.47	1069	1065[3]	0.2%	MS, *I*
2-ethyl-3,6-dimethylpyrazine (**15**)	15.87	1080	1077[4]	0.6%	MS, *I*
2-ethyl-3,5-dimethylpyrazine (**16**)	16.05	1086	1082[4]	<0.1%	MS, *I*
tetramethylpyrazine (**17**)	16.13	1088	1087[4]	0.1%	MS, *I*
2-nonanone(**69**)	16.33	1094	1090[3]	<0.1%	MS, *I*
2-phenylethylamine (**33**)	16.52	1099	1100[9]	7.5%	com, *I*
2-phenylethanol (**30**)	17.05	1116	1107[3]	13.9%	MS, *I*
phenylacetonitrile (**36**)	17.87	1142	1138[3]	<0.1%	MS, *I*
benzothiazole (**26**)	20.57	1230	1246[5]	0.1%	MS, *I*
3-methyl-2-decanone (**84**)	21.02	1245		0.2%	syn
2-butyl-3,5-dimethylpyrazine (**18**)	21.42	1259	1263[4]	0.1%	MS, *I*
2-phenylethyl acetate (**31**)	21.46	1260	1254[3]	0.1%	MS, *I*
2-undecanone (**70**)	22.47	1295	1293[3]	<0.1%	MS, *I*
indole (**27**)	22.55	1297	1290[3]	<0.1%	MS, *I*
o-aminoacetophenone (**42**)	22.78	1306	1299[10]	0.2%	com, *I*
2-(3-methylbutyl)-3,5-dimethylpyrazine (**19**)	23.12	1318	1321[4]	0.1%	MS, *I*
benzamide (**43**)	23.52	1333	1339[11]	<0.1%	MS, *I*
3-methyl-2-undecanone (**85**)	23.89	1347	1342[12]	0.2%	syn

4-methylquinazoline (**28**)	23.98	1350	1363[13]	<0.1%	MS, *I*
4-methylquinoline (**29**)	24.93	1385	1399[5]	<0.1%	MS, *I*
3-methyl-*N*-(2-phenylethylidene)-1-butanamine (**67**)	25.03	1388	1387[14]	0.1%	syn
phenylacetamide (**44**)	25.25	1396	1393[15]	0.3%	MS, *I*
N-(3-methylbutylidene)-2-phenylethylamine (**62**)	26.13	1430		0.1%	syn
3-methyl-2-dodecanone (**86**)	26.57	1448	1443[12]	<0.1%	syn
N-(2-phenylethyl)pyrrole (**56**)	26.66	1451		0.2%	syn
geranyl acetone (**2**)	26.79	1456	1453[3]	0.2%	MS, *I*
N-(2-phenylethyl)formamide (**46**)	27.66	1490		8.8%	syn
N-(2-phenylethyl)acetamide (**47**)	28.31	1516		3.2%	syn
2-methyl-*N*-(2-phenylethyl)pyrrole (**58**)	29.05	1547		0.4%	syn
12-methyl-2-tridecanone (**75**)	29.42	1562	1569[16]	<0.1%	MS, *I*
nerolidol (**4**)	29.57	1568	1561[3]	0.1%	MS, *I*
N-(2-phenylethyl)propanamide (**48**)	30.21	1594		0.1%	syn
2-tetradecanone (**71**)	30.31	1599	1597[17]	0.1%	MS
benzophenone (**41**)	31.25	1640	1626[3]	0.1%	MS, *I*
N-(2-furylmethylidene)-2-phenylethylamine (**64**)	31.33	1643		0.8%	syn
2,5-dimethyl-*N*-(2-phenylethyl)pyrrole (**60**)	31.41	1647		<0.1%	syn
N-(2-phenylethyl)butanamide (**49**)	32.20	1681		0.2%	syn
2-pentadecanone (**72**)	32.64	1701	1697[3]	0.2%	MS, *I*
N-(2-phenylethyl)-3-methylbutanamide (**53**)	33.23	1728		0.1%	syn
13-methyl-2-tetradecanone (**76**)	34.06	1766	1770[16]	0.1%	MS, *I*
N-(2-phenylethyl)pentanamide (**50**)	34.45	1784	1795	0.1%	syn
2-hexadecanone (**73**)	34.85	1802	1800[17]	0.1%	MS
N-benzylidene-2-phenylethylamine (**65**)	34.92	1806	1818	10.9%	syn
diphenylethanedione (**39**)	35.34	1826		0.1%	MS
6,10,14-trimethyl-2-pentadecanone (**5**)	35.80	1848	1847[16]	<0.1%	MS, *I*
2-phenylethyl benzoate (**32**)	36.22	1868	1859[9]	0.2%	syn
N-(2-phenylethyl)hexanamide (**51**)	36.63	1888		0.1%	syn
2-heptadecanone (**74**)	36.95	1904	1902[17]	<0.1%	MS
farnesylacetone (**3**)	37.36	1925	1913[3]	1.2%	MS, *I*
N-(2-phenylethyl)octanamide (**52**)	40.72	2099		0.2%	syn
N-(2-phenylethyl)benzamide (**54**)	40.91	2109		4.0%	syn

[a] *I* = retention index, Ident. = Identification by: MS = mass spectrum, com = commercial sample, syn = synthetic sample.

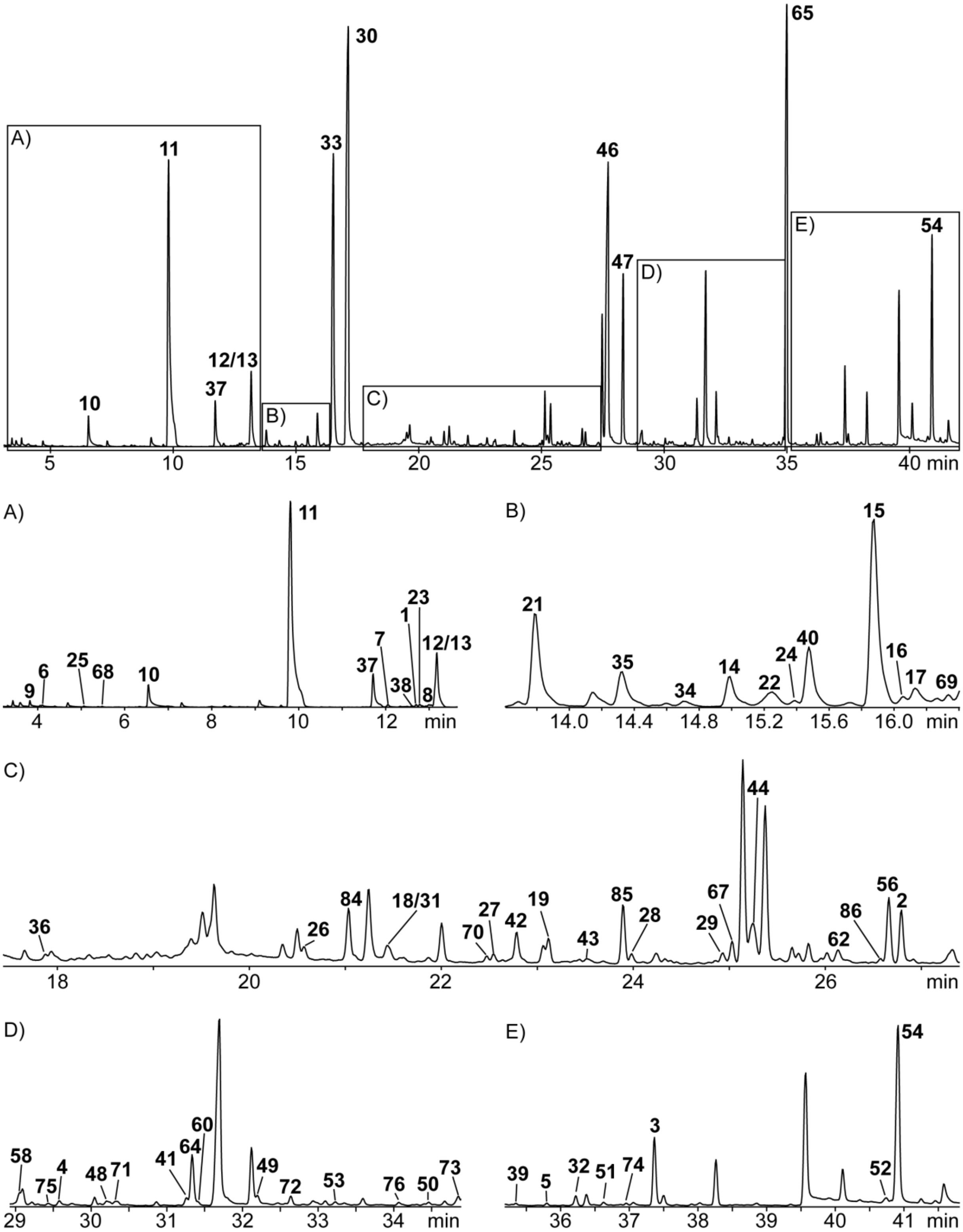

Figure S1. Total ion chromatogram of a headspace extract obtained from CLSA analysis of *C. polytrichastri*. Parts A) – E) show expansions for partial chromatograms in boxes.

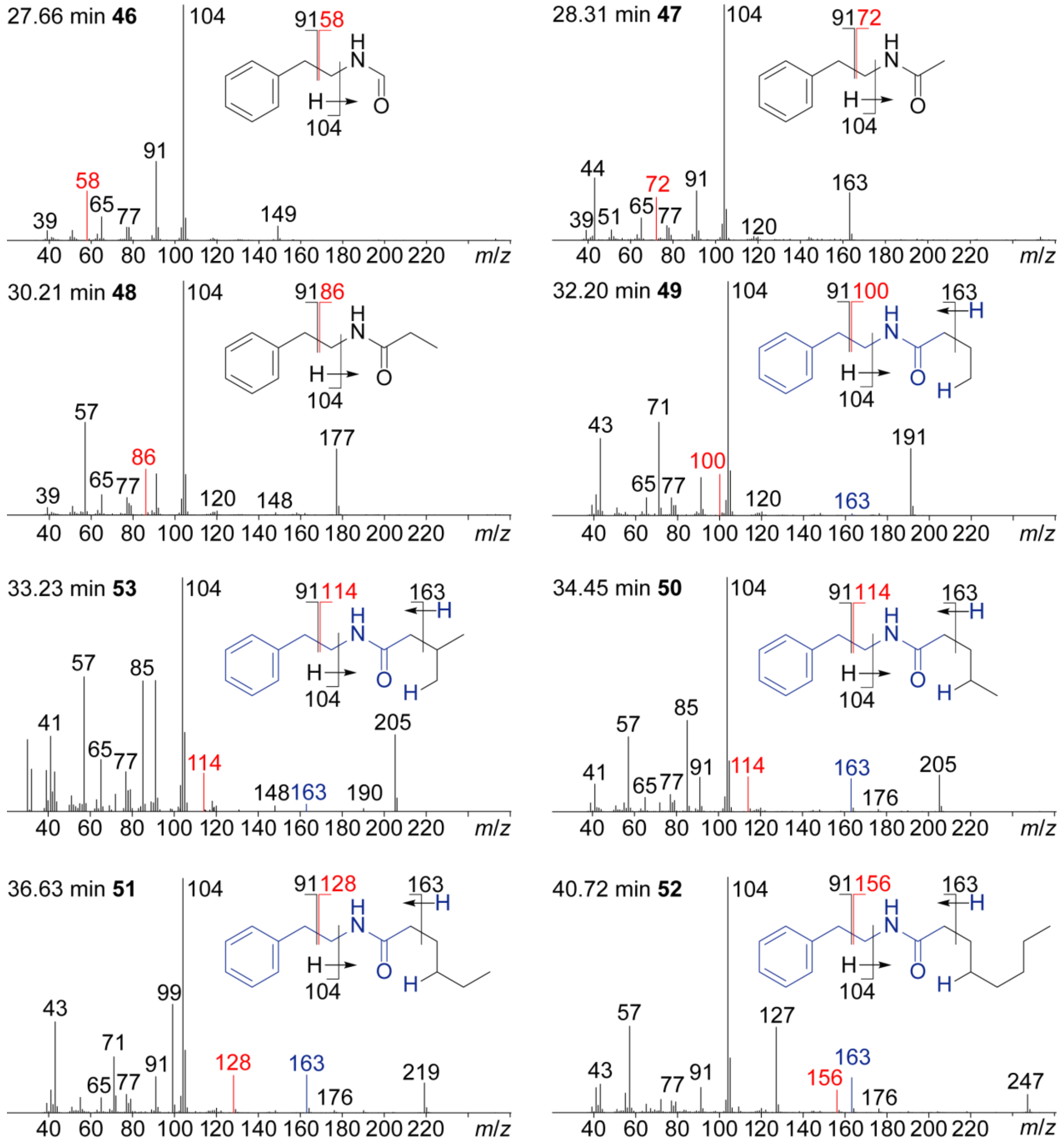

Figure S2. EI mass spectra of *N*-(2-phenylethyl)amides **46** – **52**. Red fragments arise from cleavage of the benzyl group, blue fragments indicate McLafferty rearrangement.

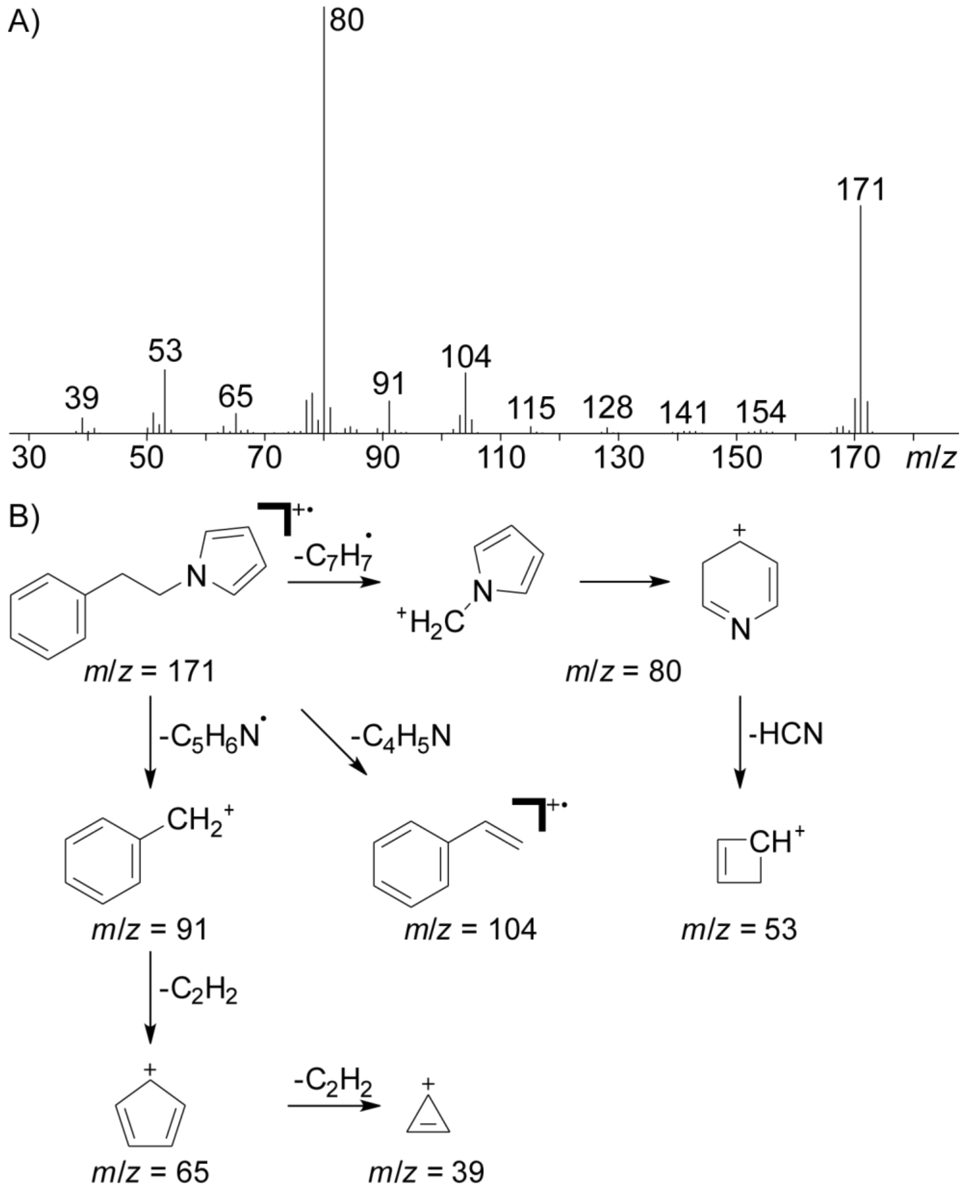

Scheme S1. A) EI-MS spectrum of **56**. B) Fragmentation mechanism explaining the most abundant fragment ions used for the structural proposal.

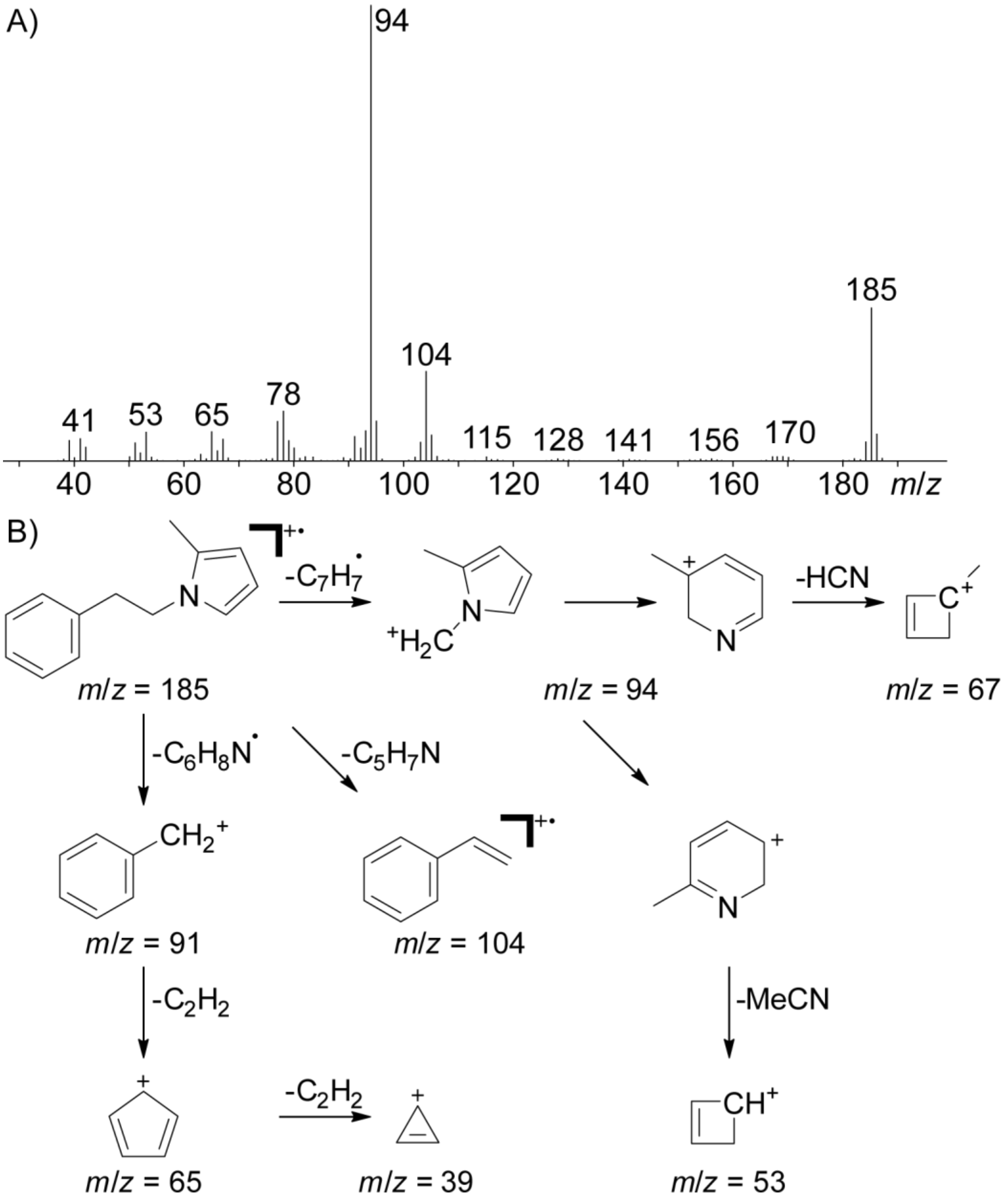

Scheme S2. A) EI-MS spectrum of **58**. B) Fragmentation mechanism explaining the most abundant fragment ions used for the structural proposal.

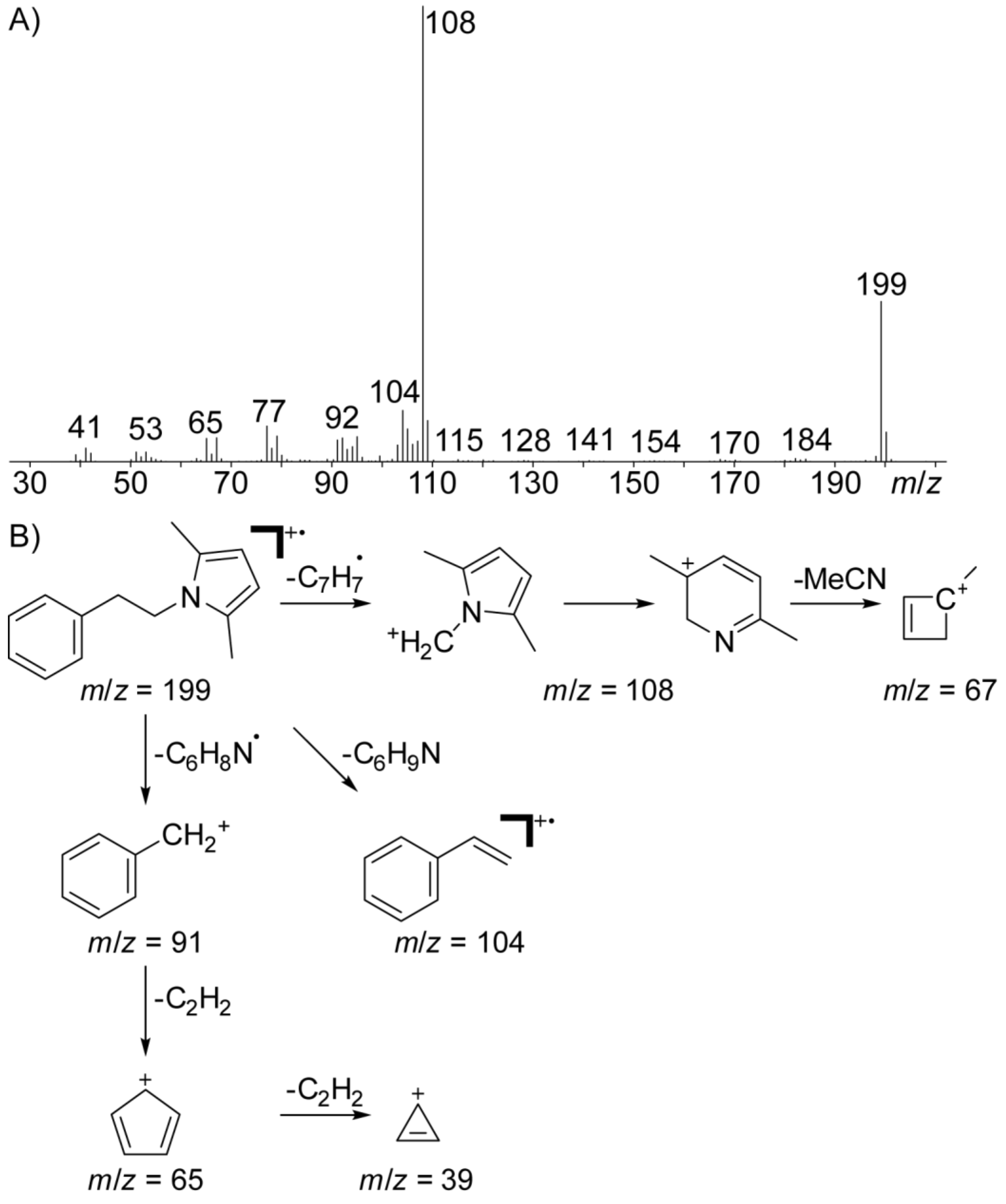

Scheme S3. A) EI-MS spectrum of **60**. B) Fragmentation mechanism explaining the most abundant fragment ions used for the structural proposal.

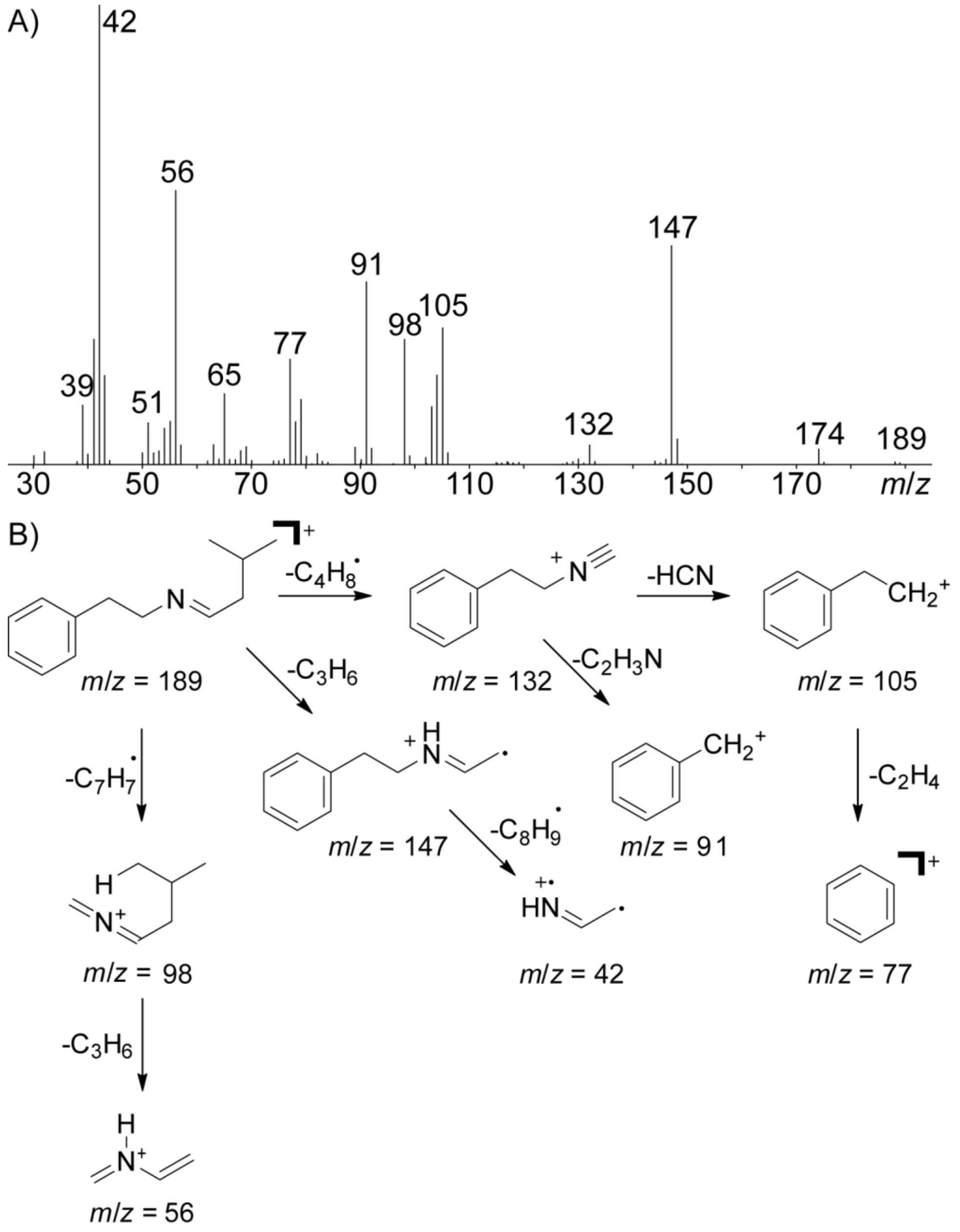

Scheme S4. A) EI-MS spectrum of **62**. B) Fragmentation mechanism explaining the most abundant fragment ions used for the structural proposal.

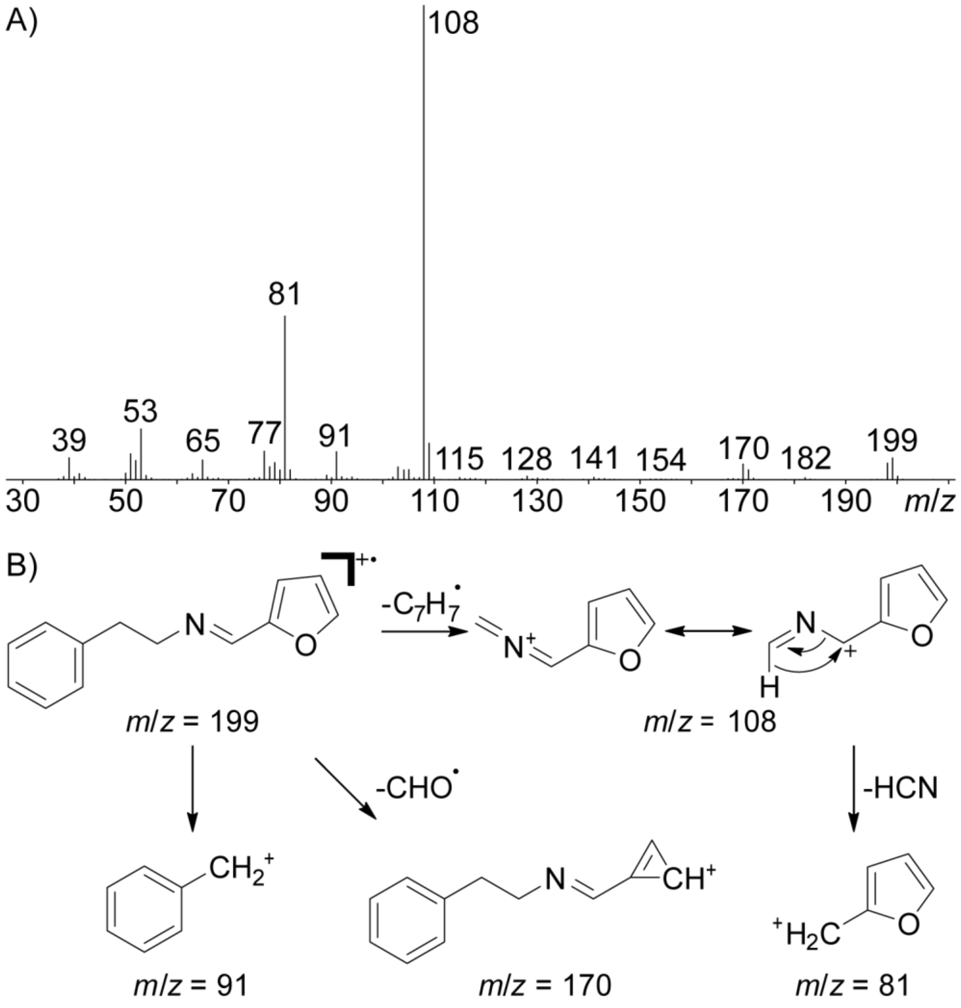

Scheme S5. A) EI-MS spectrum of **64**. B) Fragmentation mechanism explaining the most abundant fragment ions used for the structural proposal.

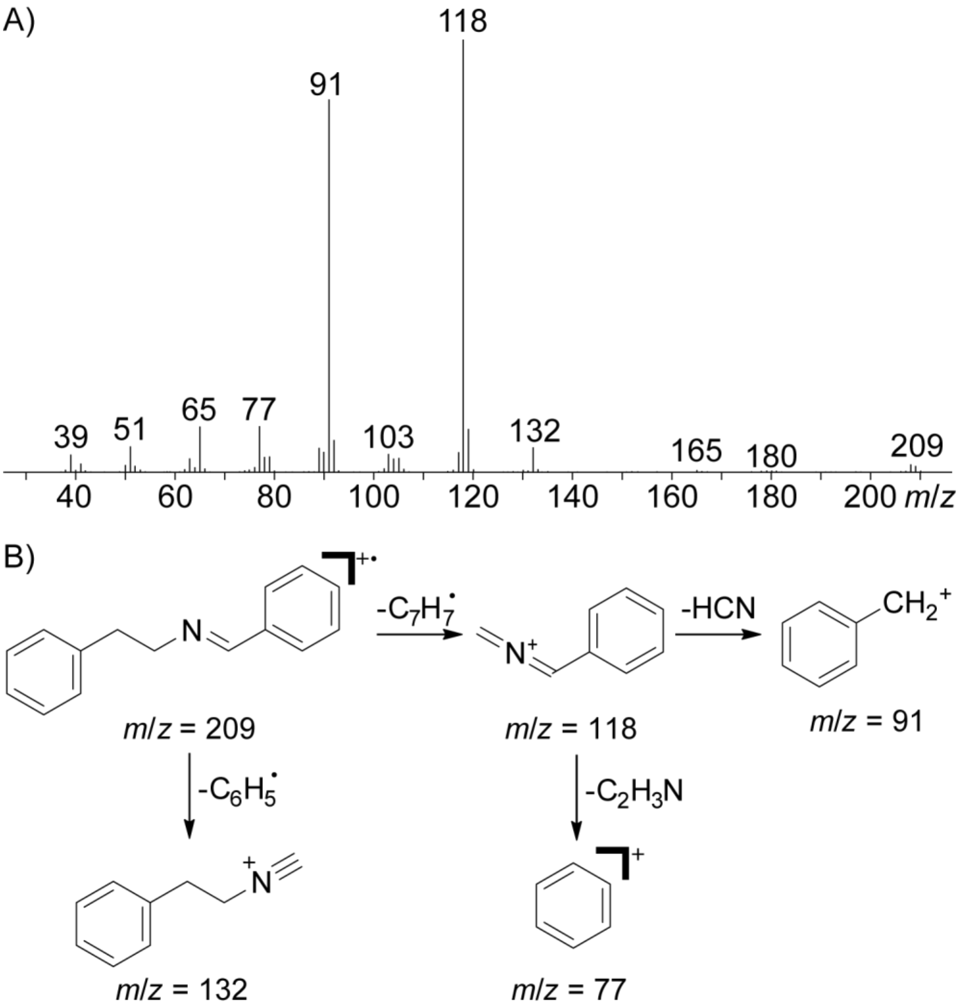

Scheme S6. A) EI-MS spectrum of **65**. B) Fragmentation mechanism explaining the most abundant fragment ions used for the structural proposal.

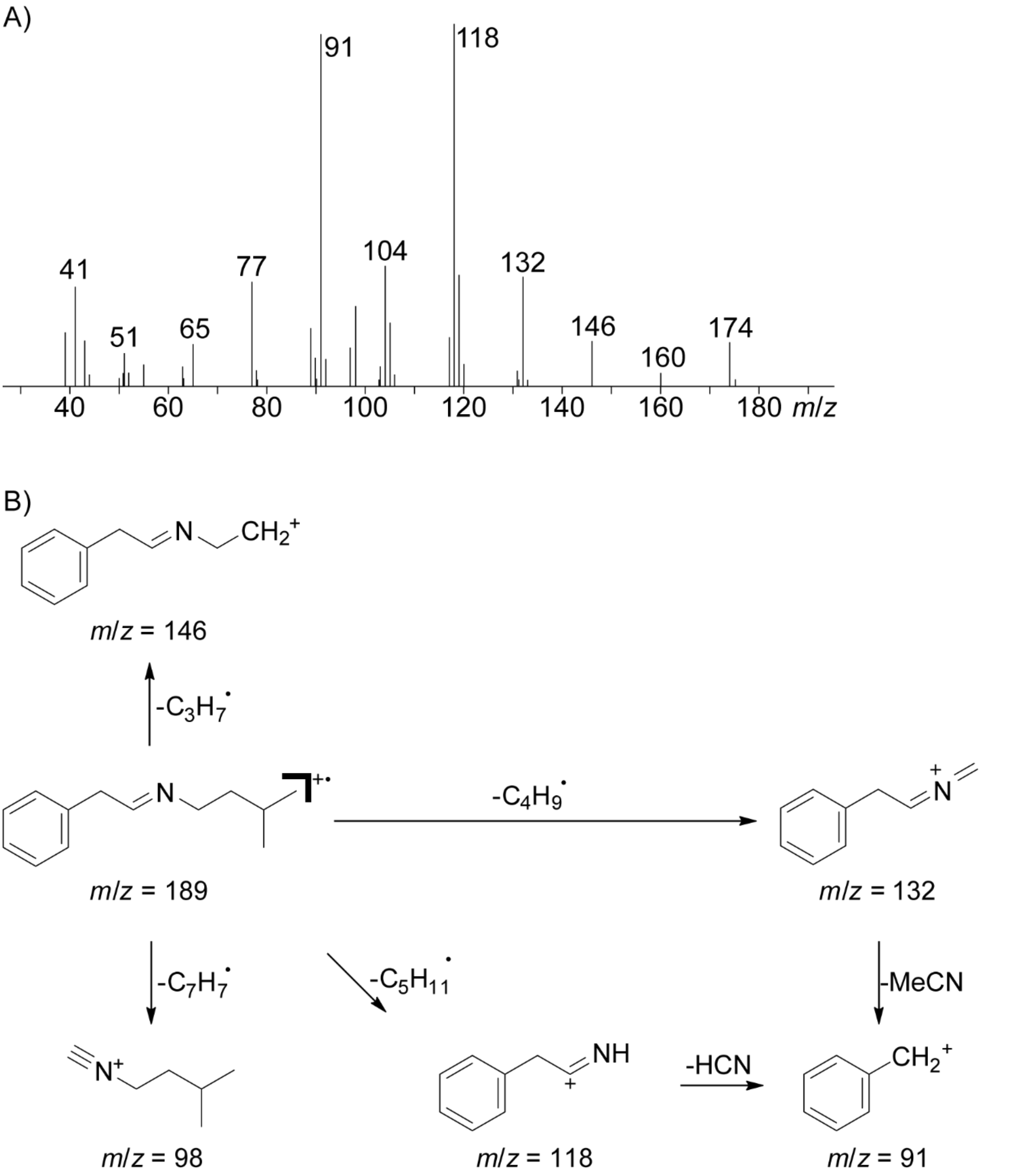

Scheme S7. A) EI-MS spectrum of **67**. B) Fragmentation mechanism explaining the most abundant fragment ions used for the structural proposal.

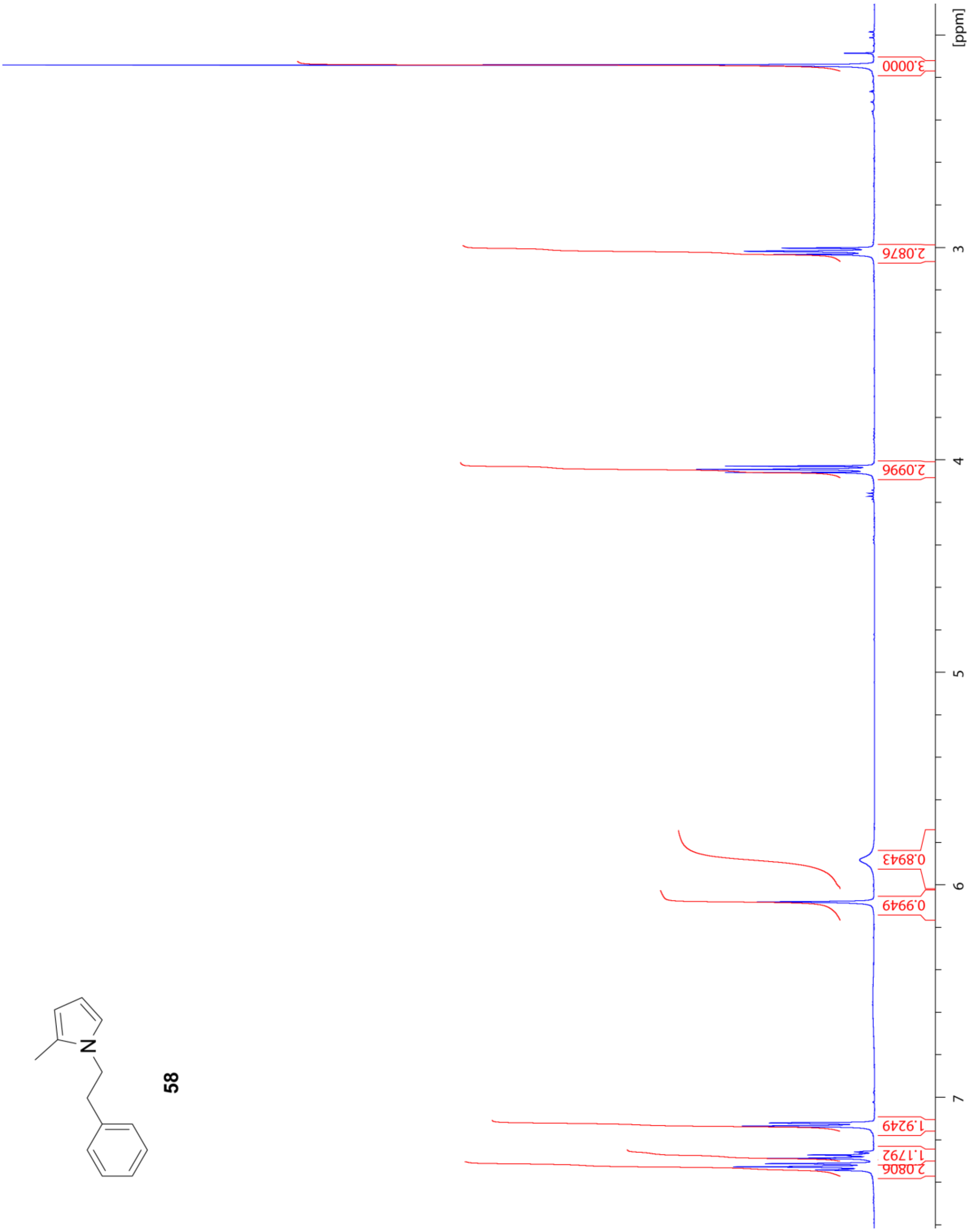

Figure S3. ^{1}H-NMR spectrum (500 MHz, $CDCl_3$) of **58**.

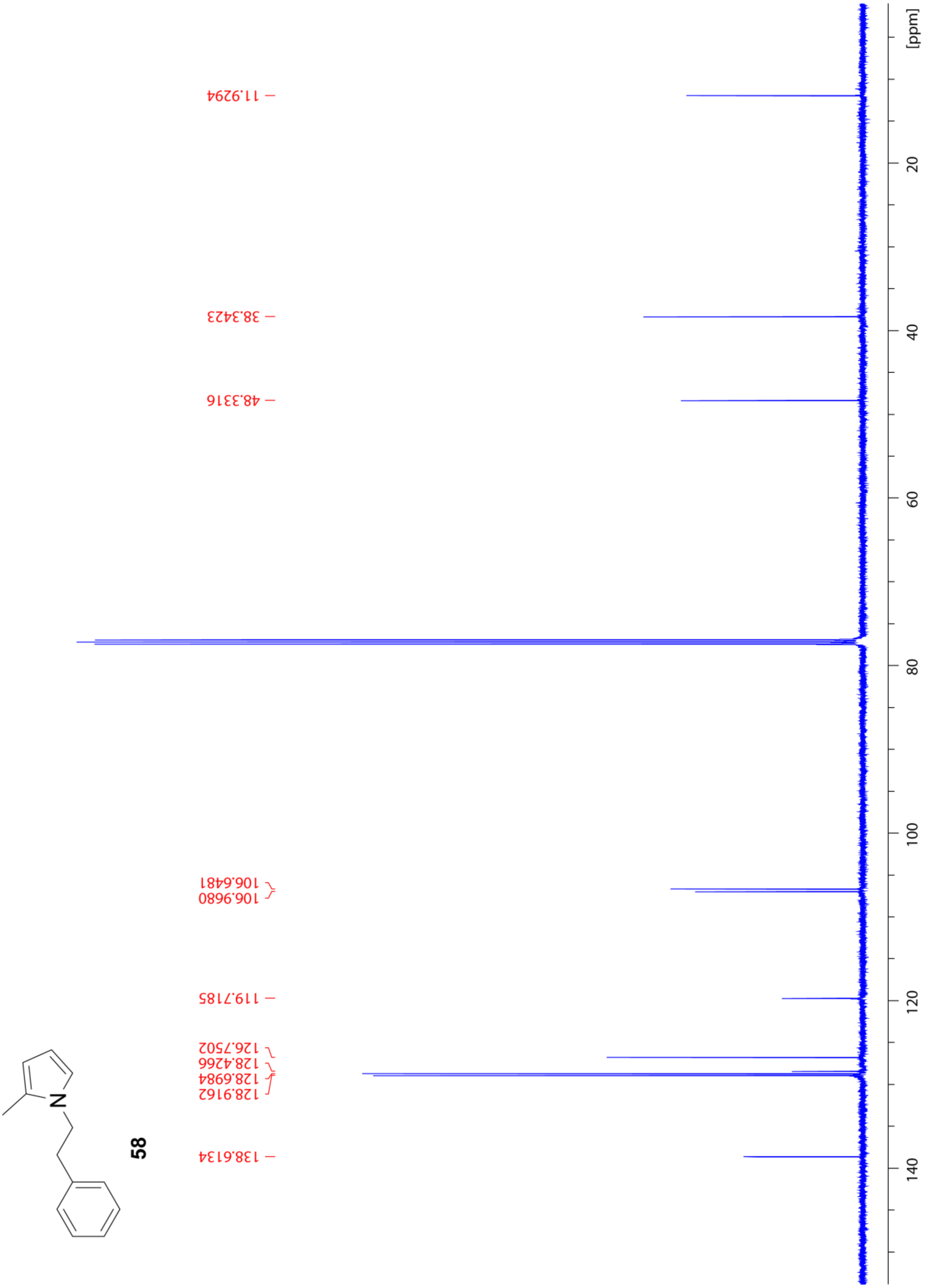

Figure S4. ^{13}C-NMR spectrum (126 MHz, $CDCl_3$) of **58**.

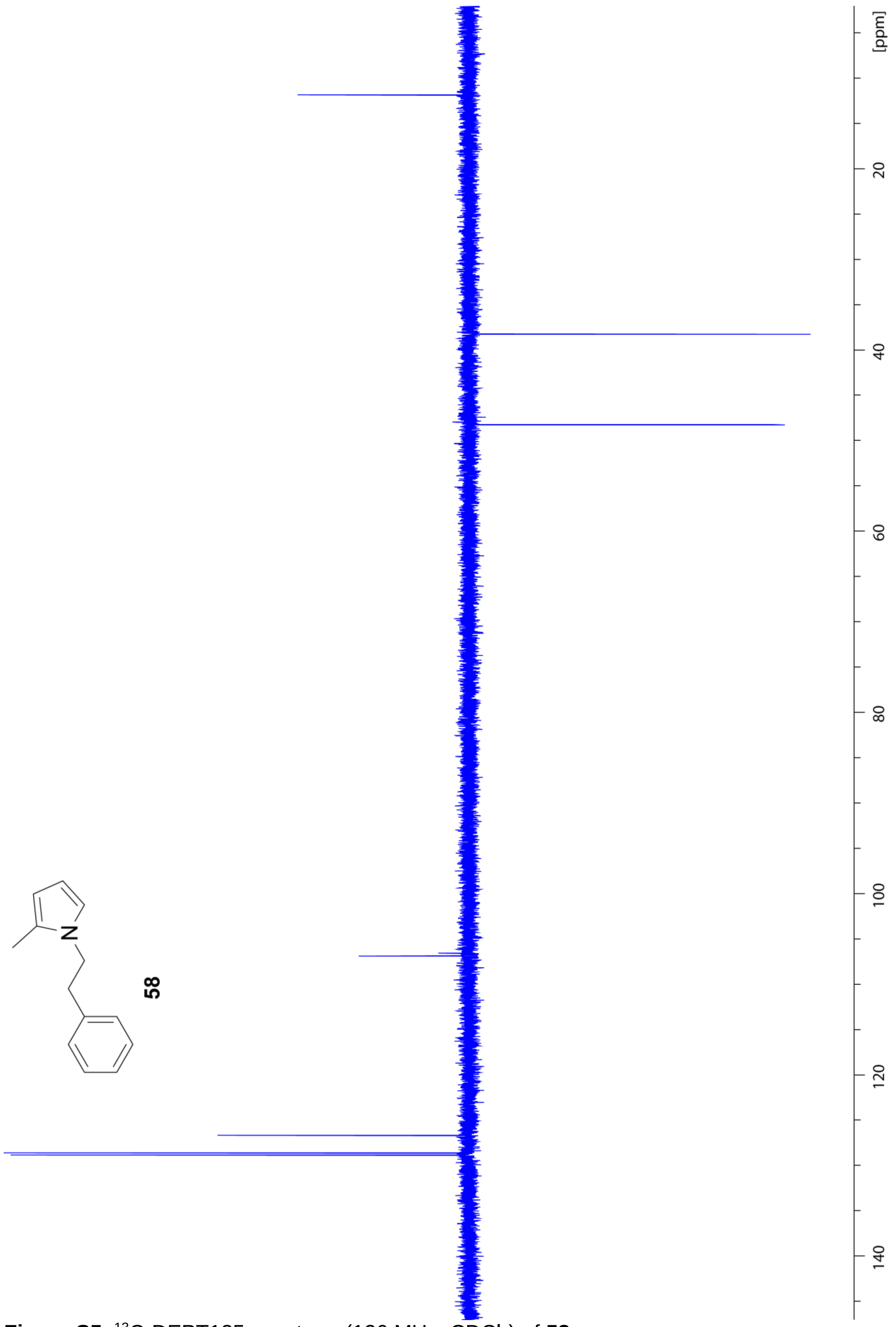

Figure S5. ^{13}C-DEPT135 spectrum (126 MHz, $CDCl_3$) of **58**.

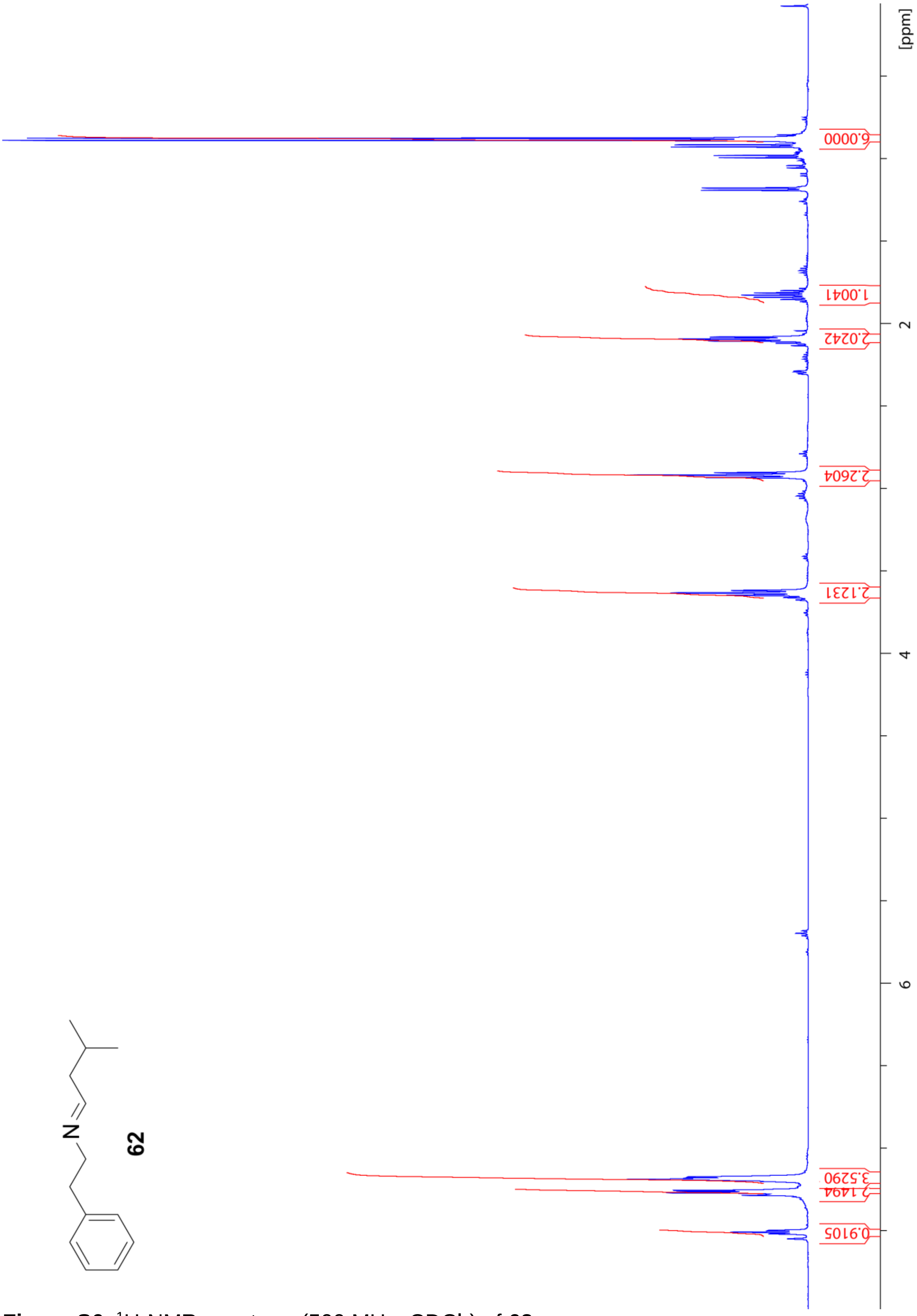

Figure S6. ^{1}H-NMR spectrum (500 MHz, $CDCl_3$) of **62**.

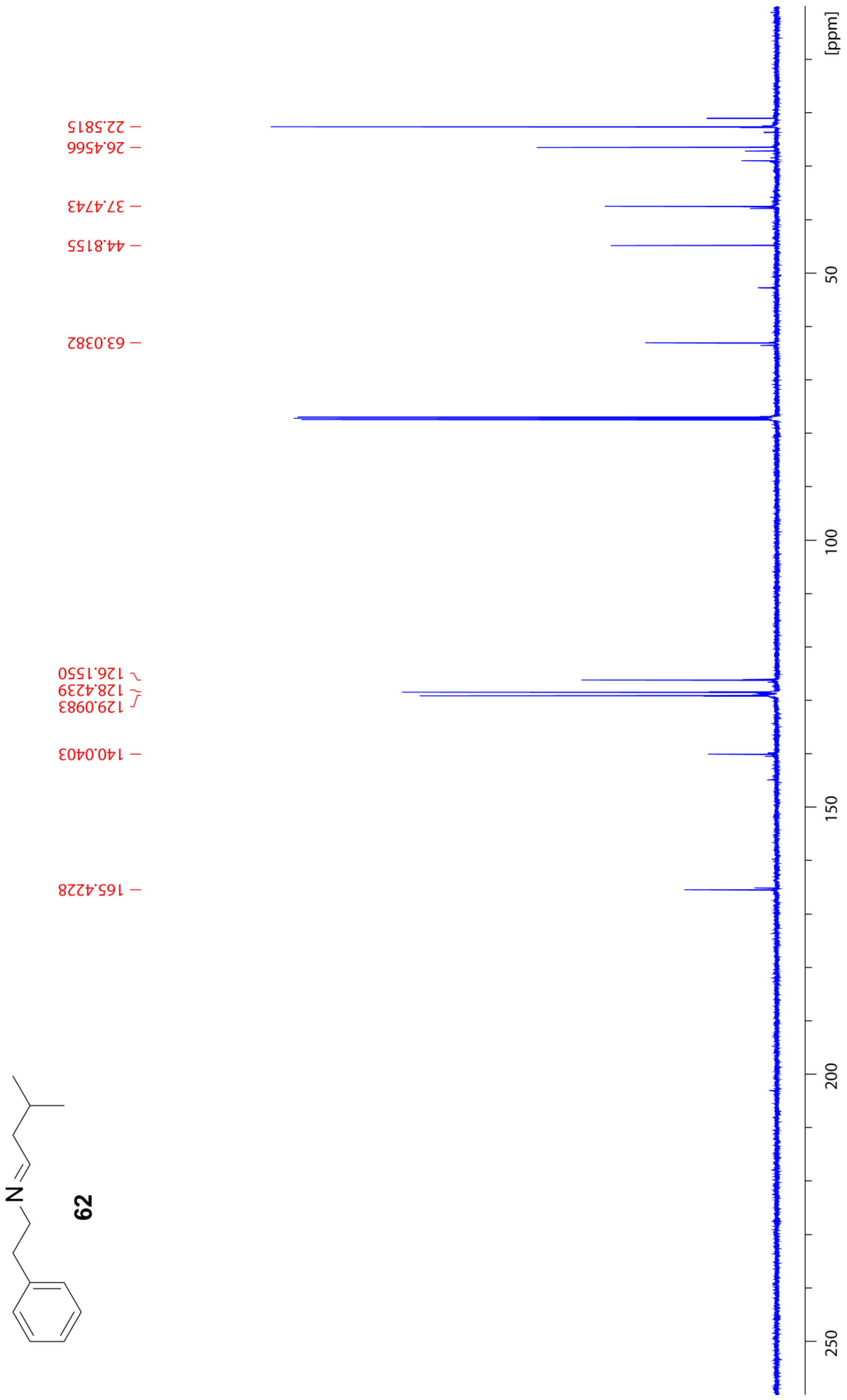

Figure S7. ^{13}C-NMR spectrum (126 MHz, $CDCl_3$) of **62**.

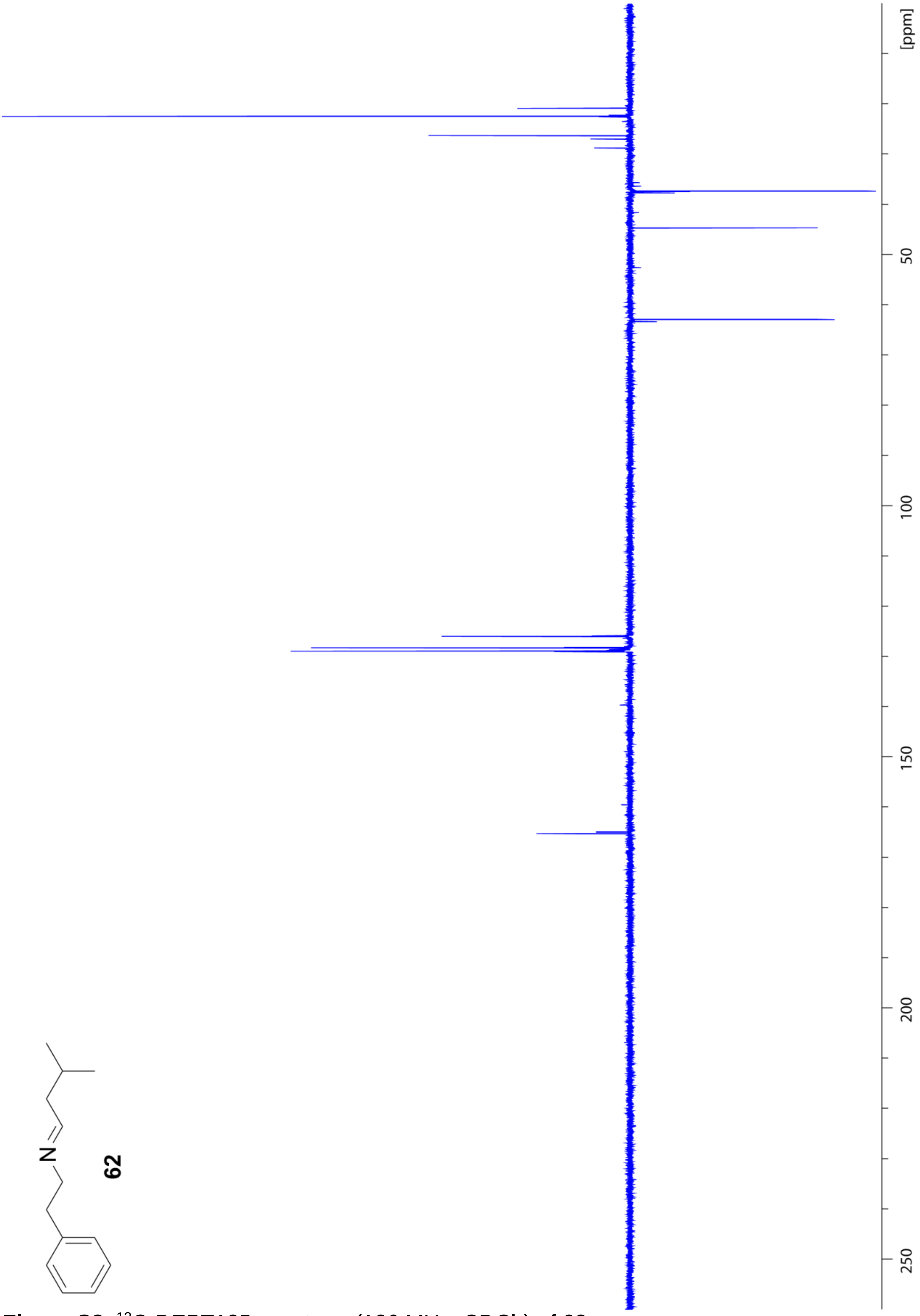

Figure S8. ^{13}C-DEPT135 spectrum (126 MHz, $CDCl_3$) of **62**.

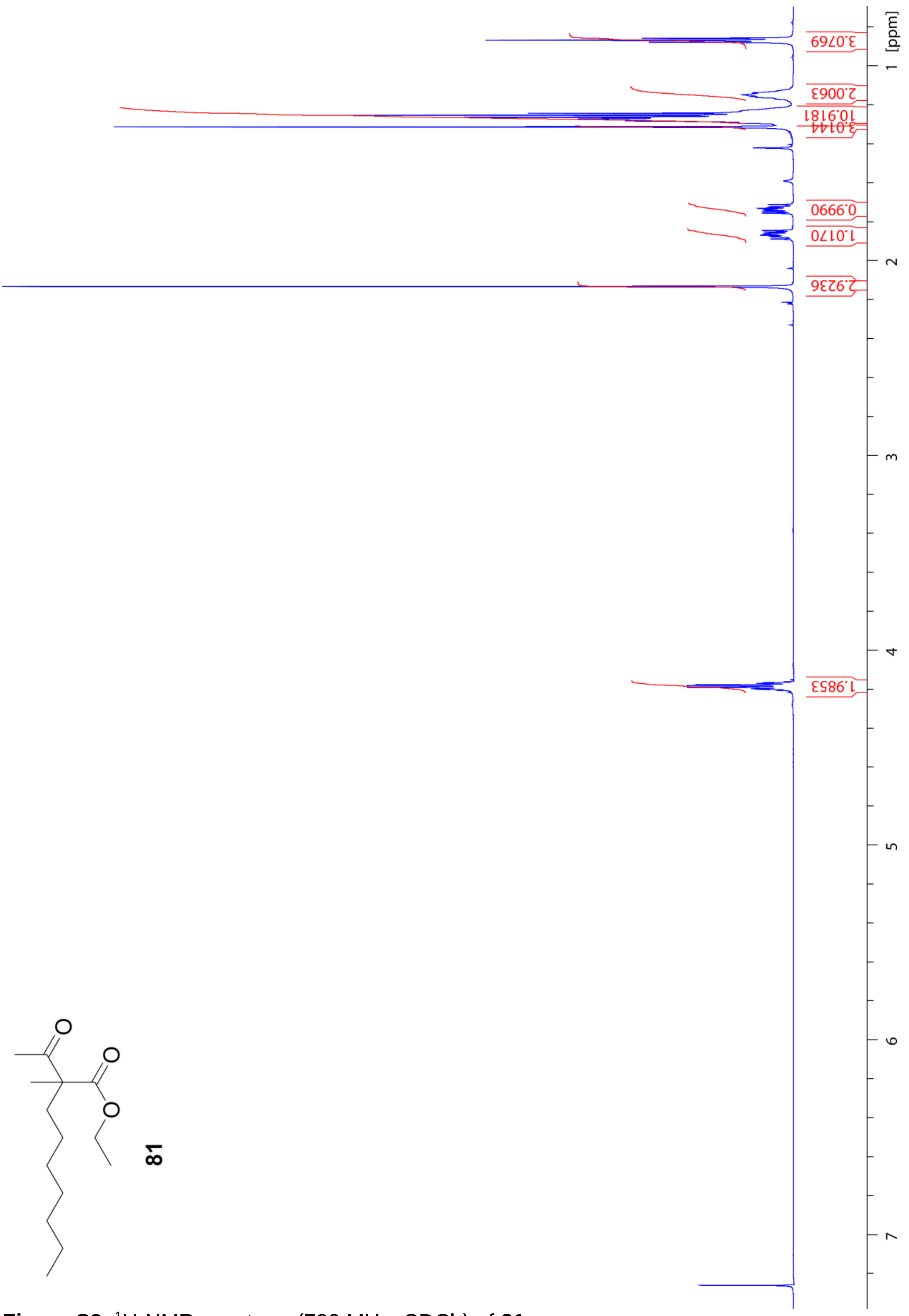

Figure S9. ^{1}H-NMR spectrum (700 MHz, $CDCl_3$) of **81**.

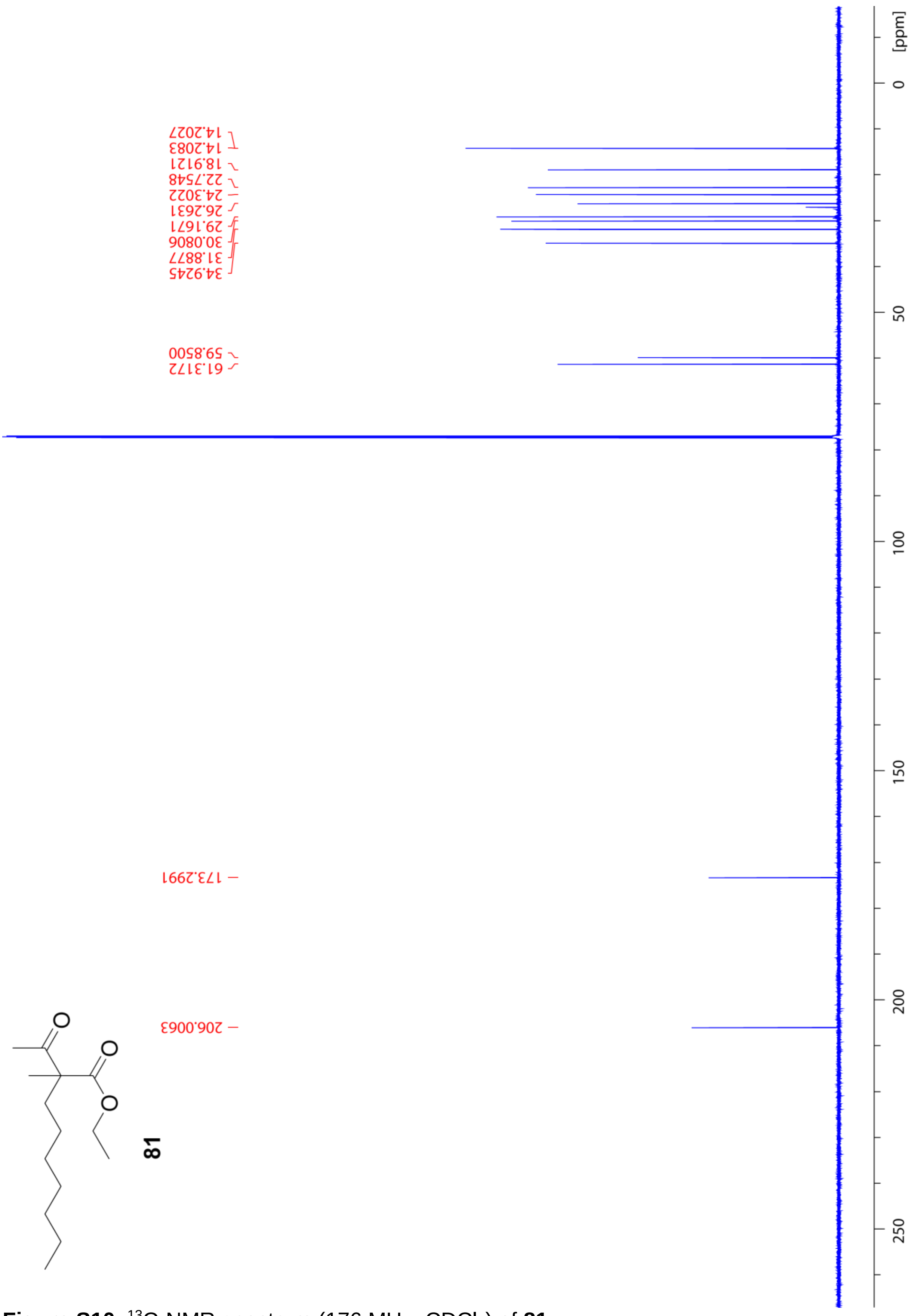

Figure S10. ^{13}C-NMR spectrum (176 MHz, $CDCl_3$) of **81**.

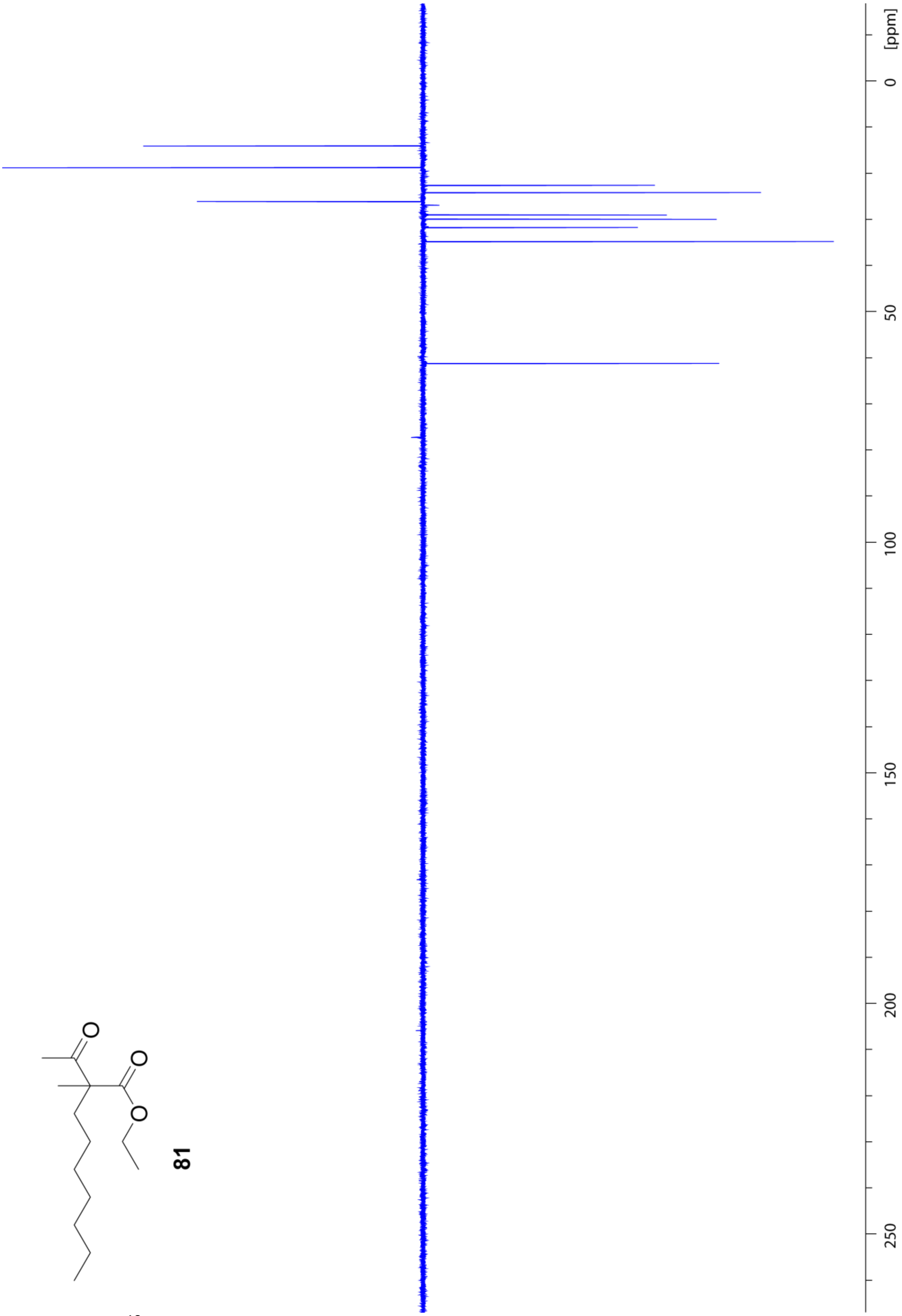

Figure S11. ^{13}C-DEPT135 spectrum (176 MHz, $CDCl_3$) of **81**.

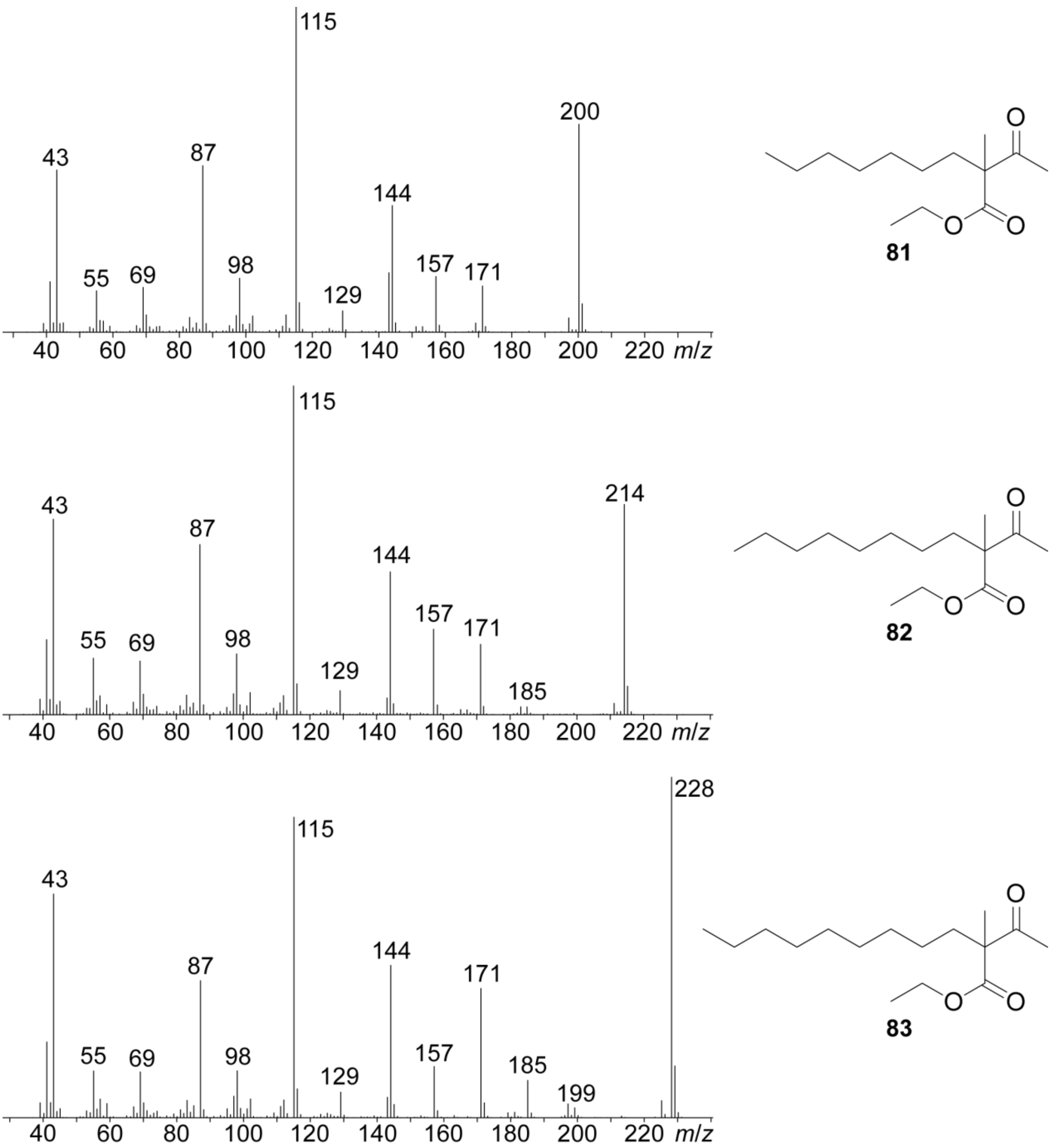

Figure S12. EI-MS spectra of β-keto esters **81 – 83**.

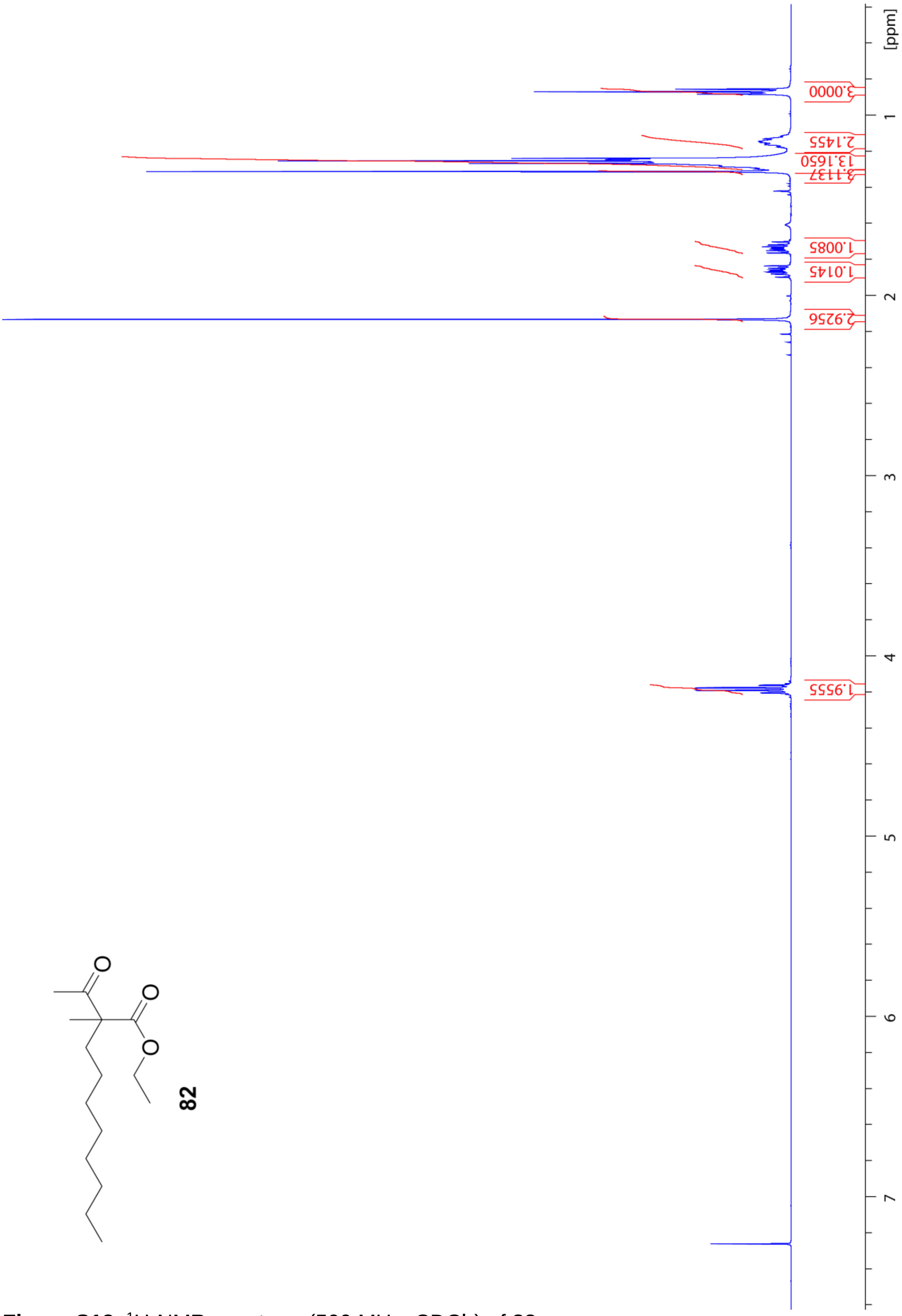

Figure S13. ^{1}H-NMR spectrum (500 MHz, $CDCl_3$) of **82**.

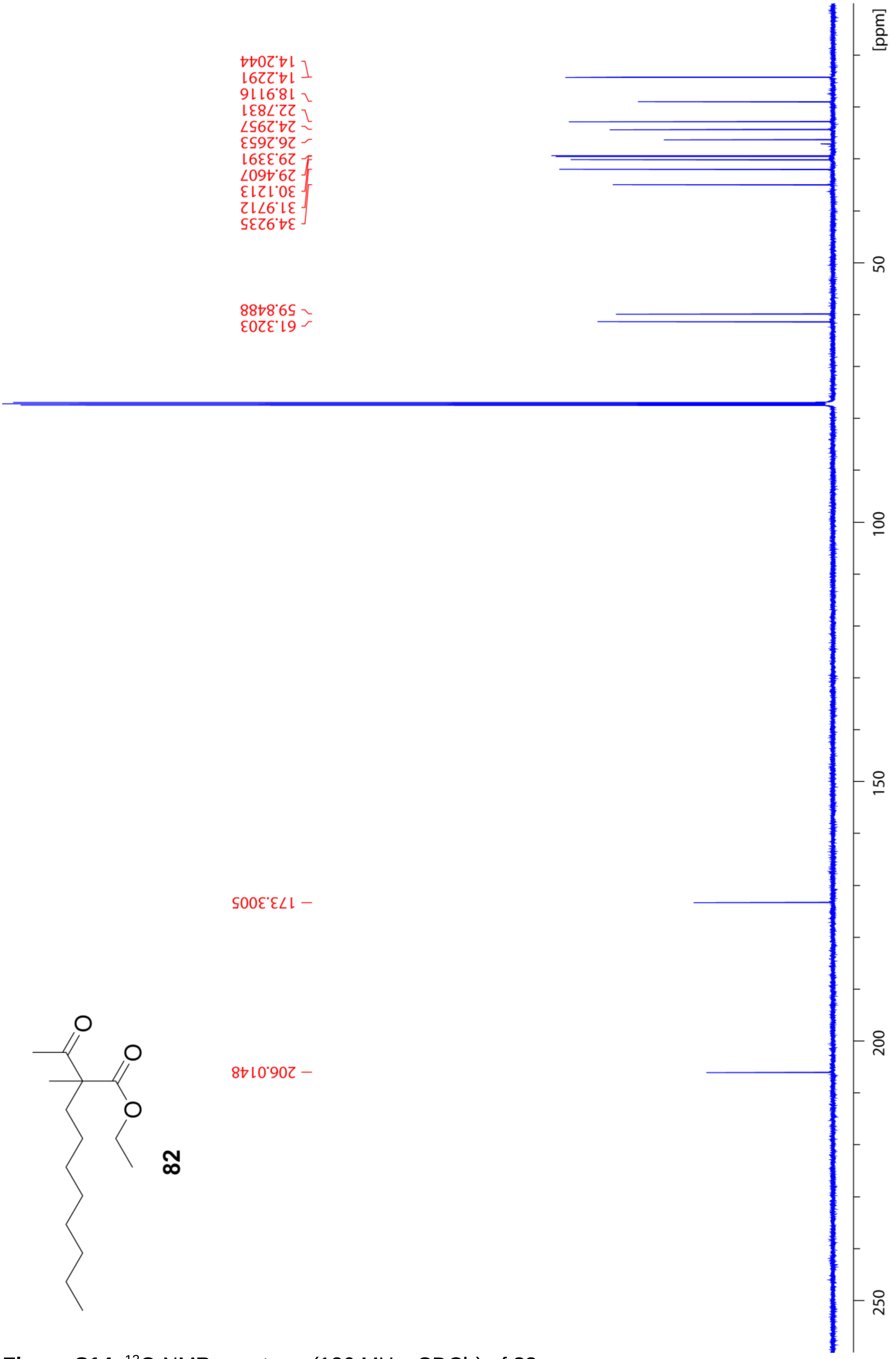

Figure S14. ^{13}C-NMR spectrum (126 MHz, $CDCl_3$) of **82**.

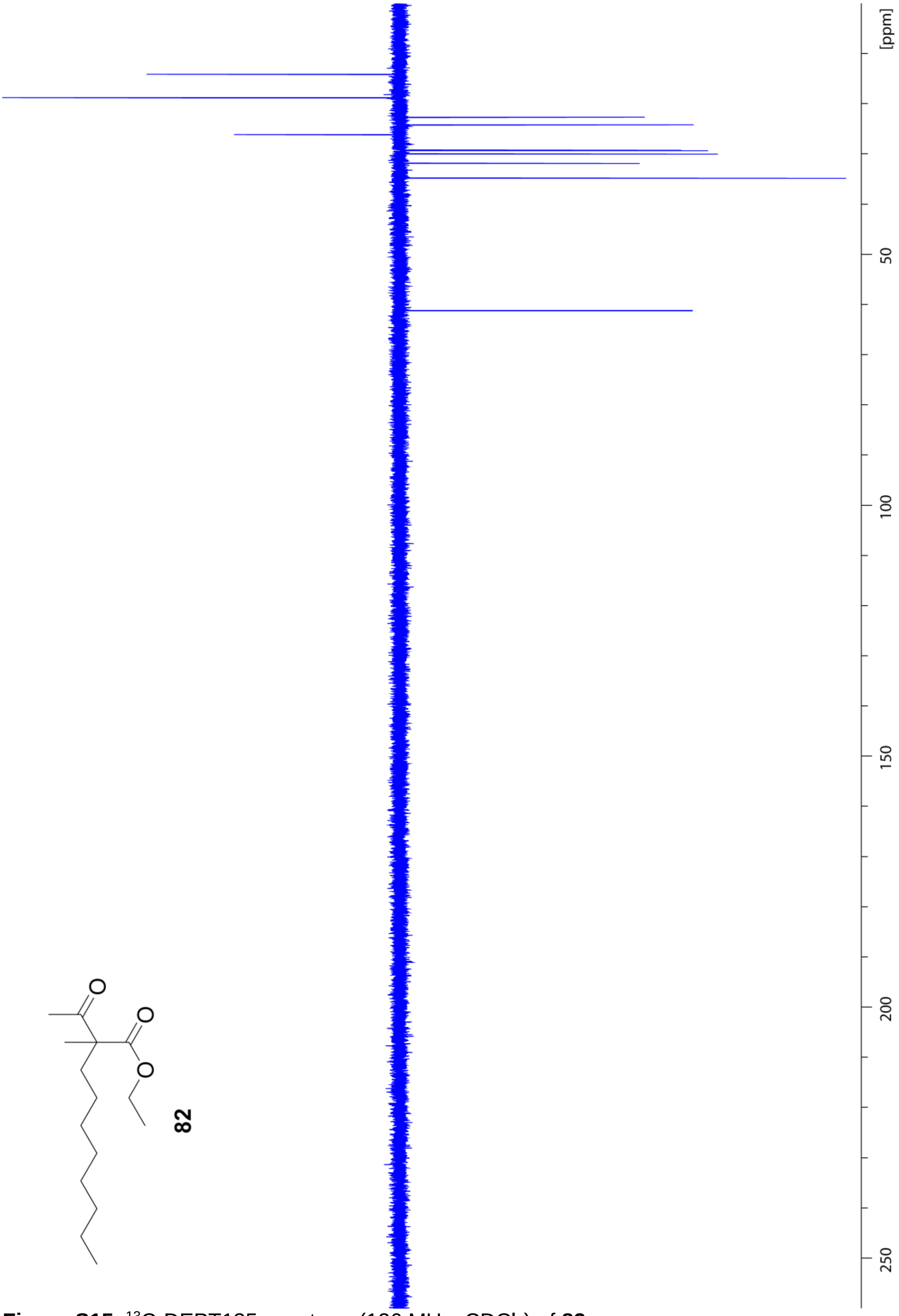

Figure S15. ^{13}C-DEPT135 spectrum (126 MHz, $CDCl_3$) of **82**.

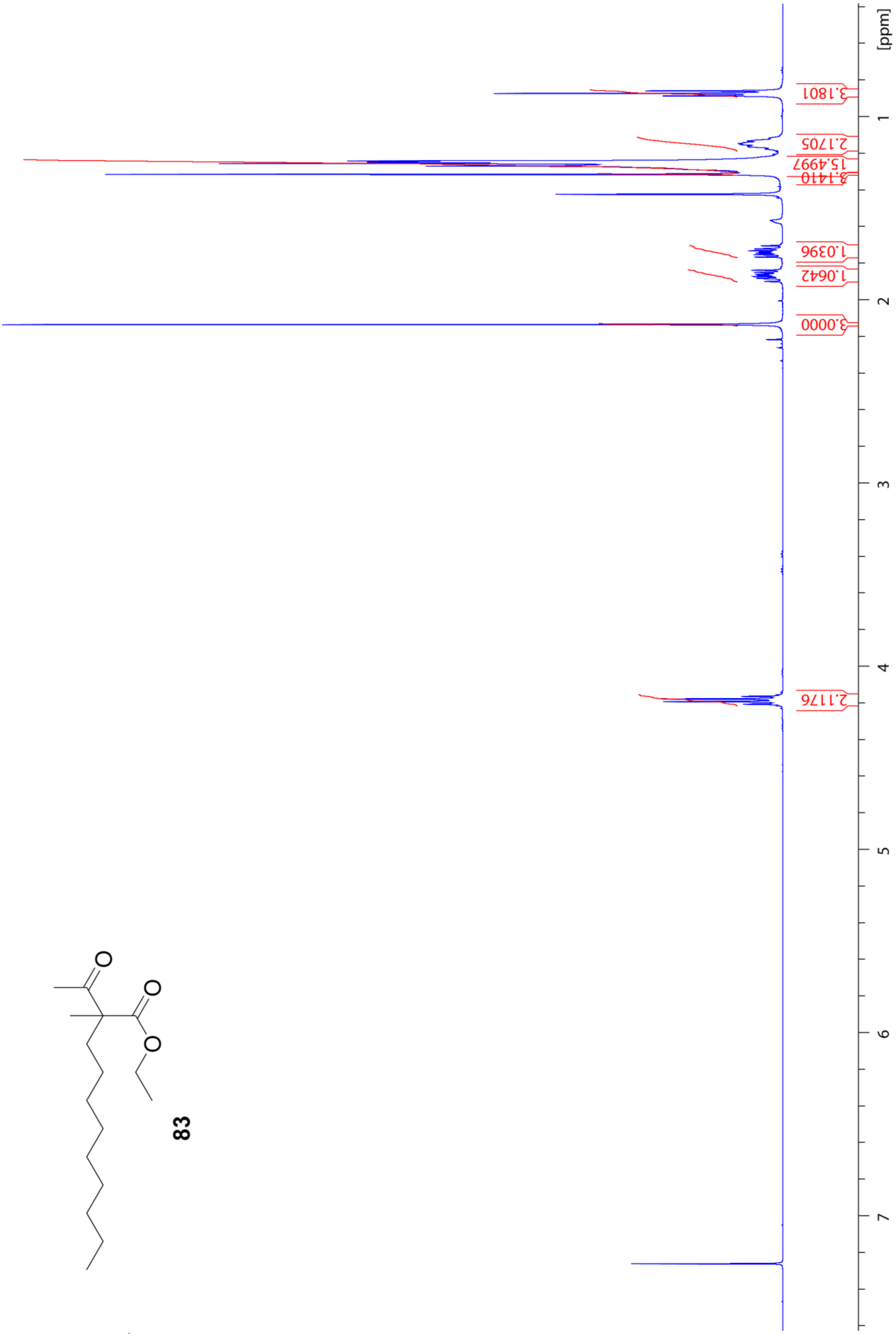

Figure S16. ^{1}H-NMR spectrum (500 MHz, $CDCl_3$) of **83**.

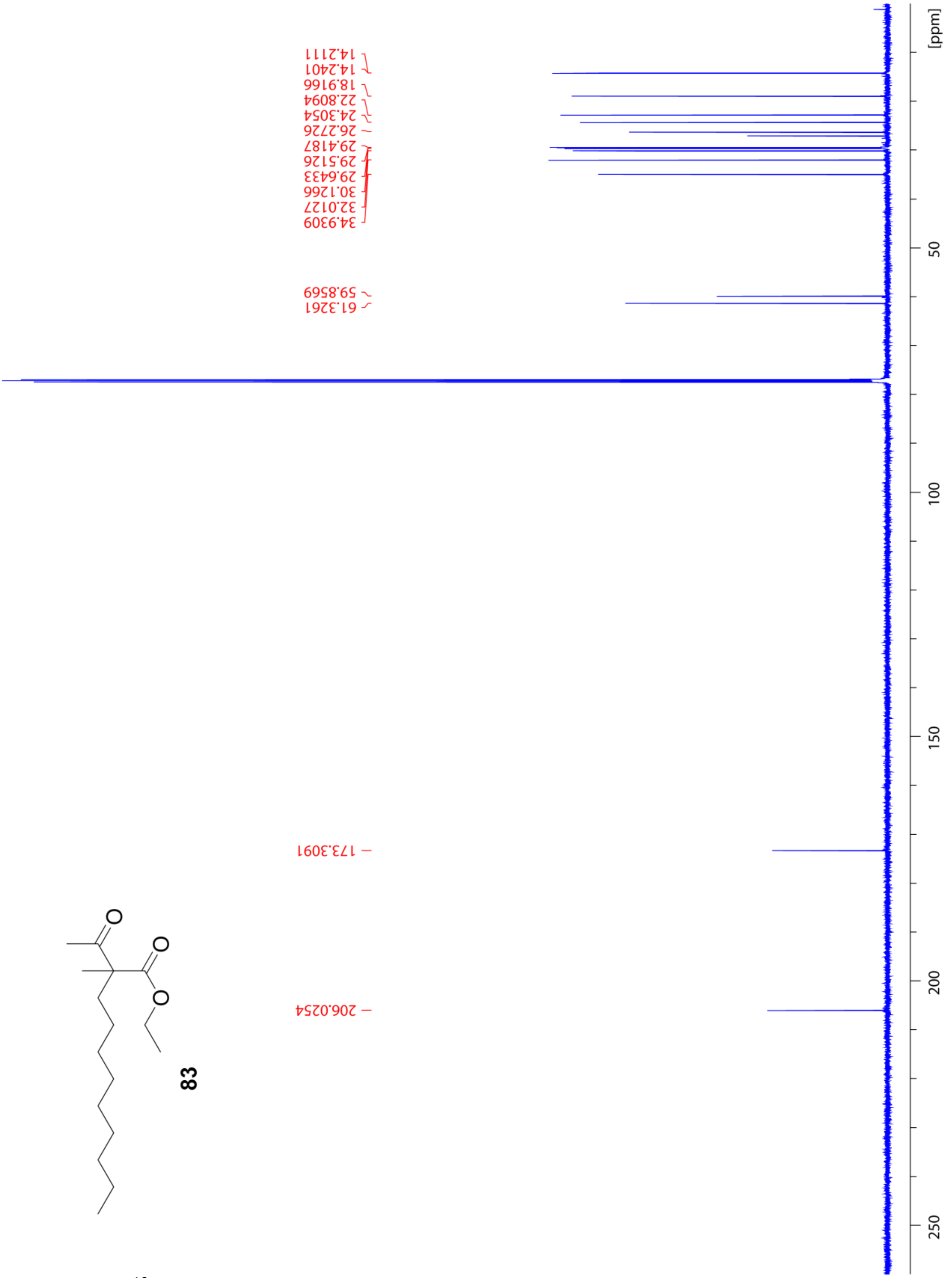

Figure S17. ^{13}C-NMR spectrum (126 MHz, $CDCl_3$) of **83**.

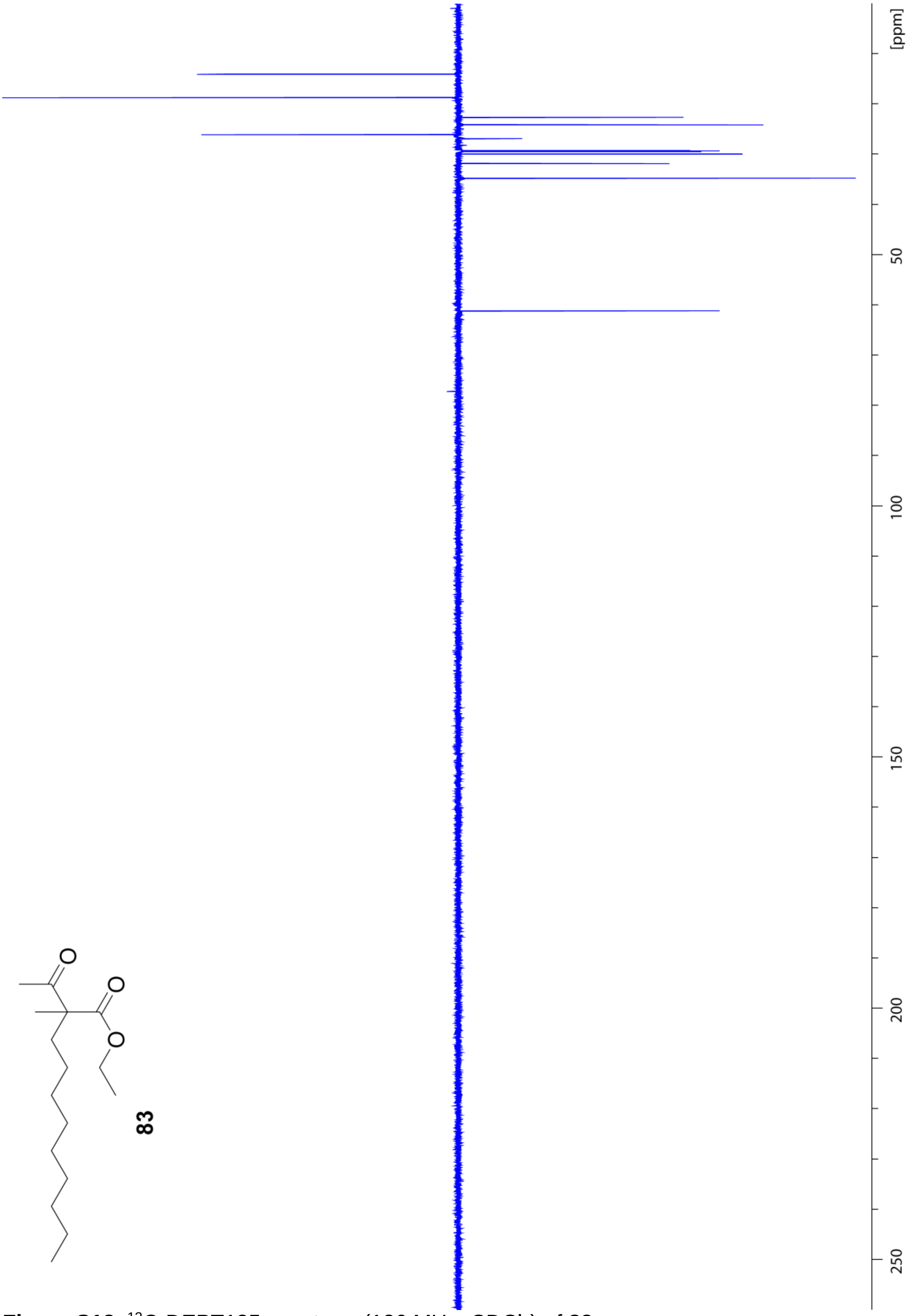

Figure S18. ^{13}C-DEPT135 spectrum (126 MHz, $CDCl_3$) of **83**.

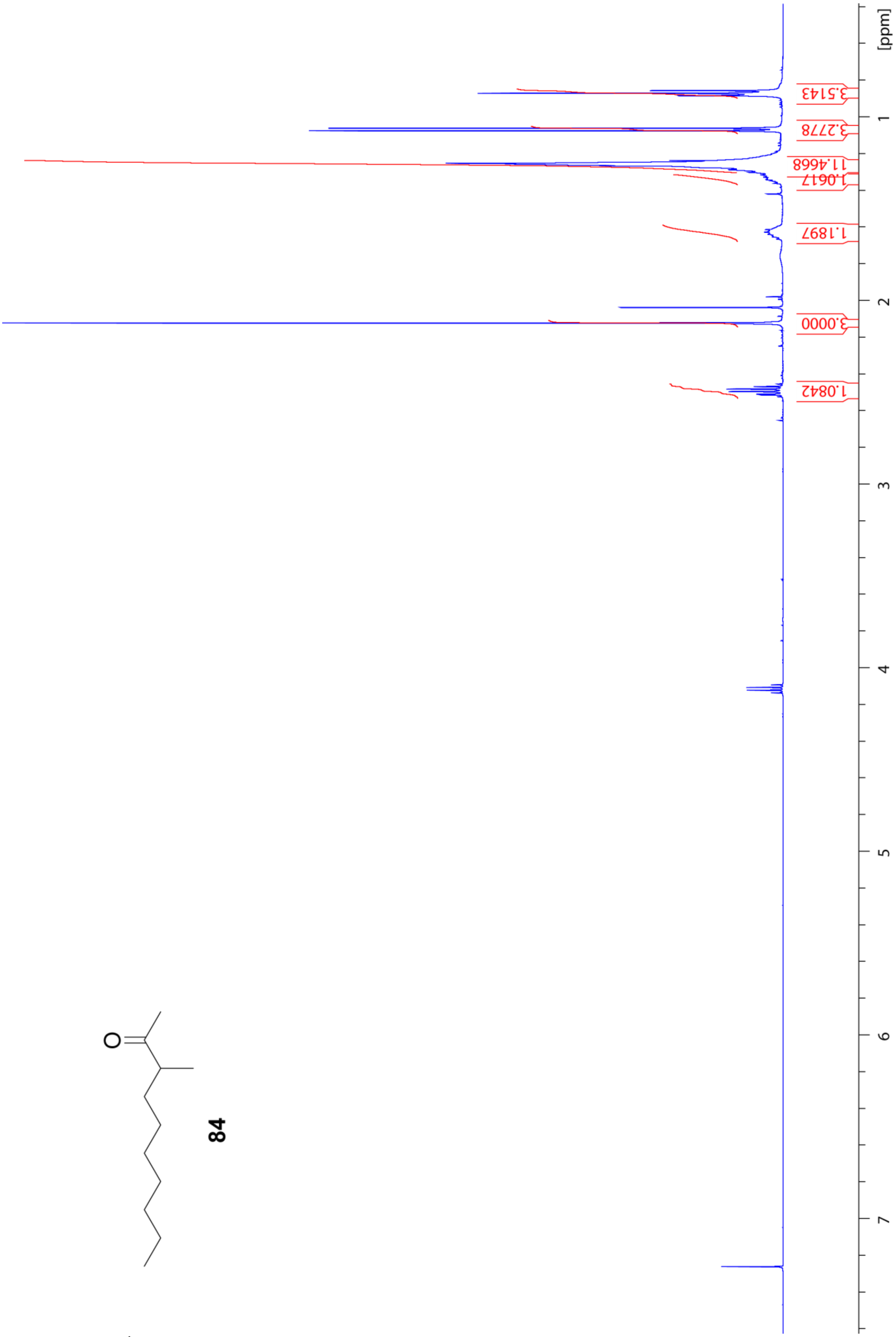

Figure S19. ^{1}H-NMR spectrum (700 MHz, $CDCl_3$) of **84**.

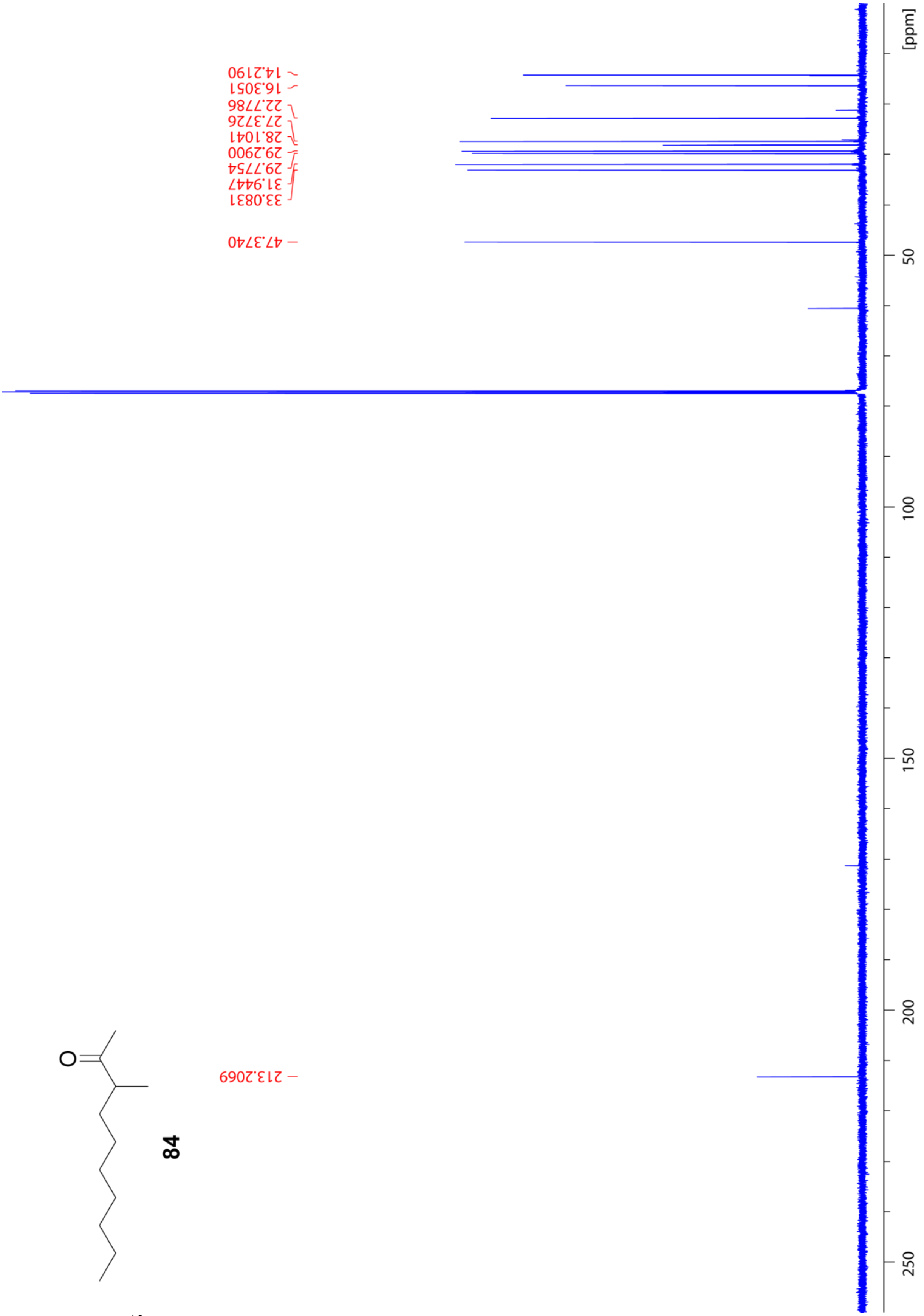

Figure S20. ^{13}C-NMR spectrum (176 MHz, $CDCl_3$) of **84**.

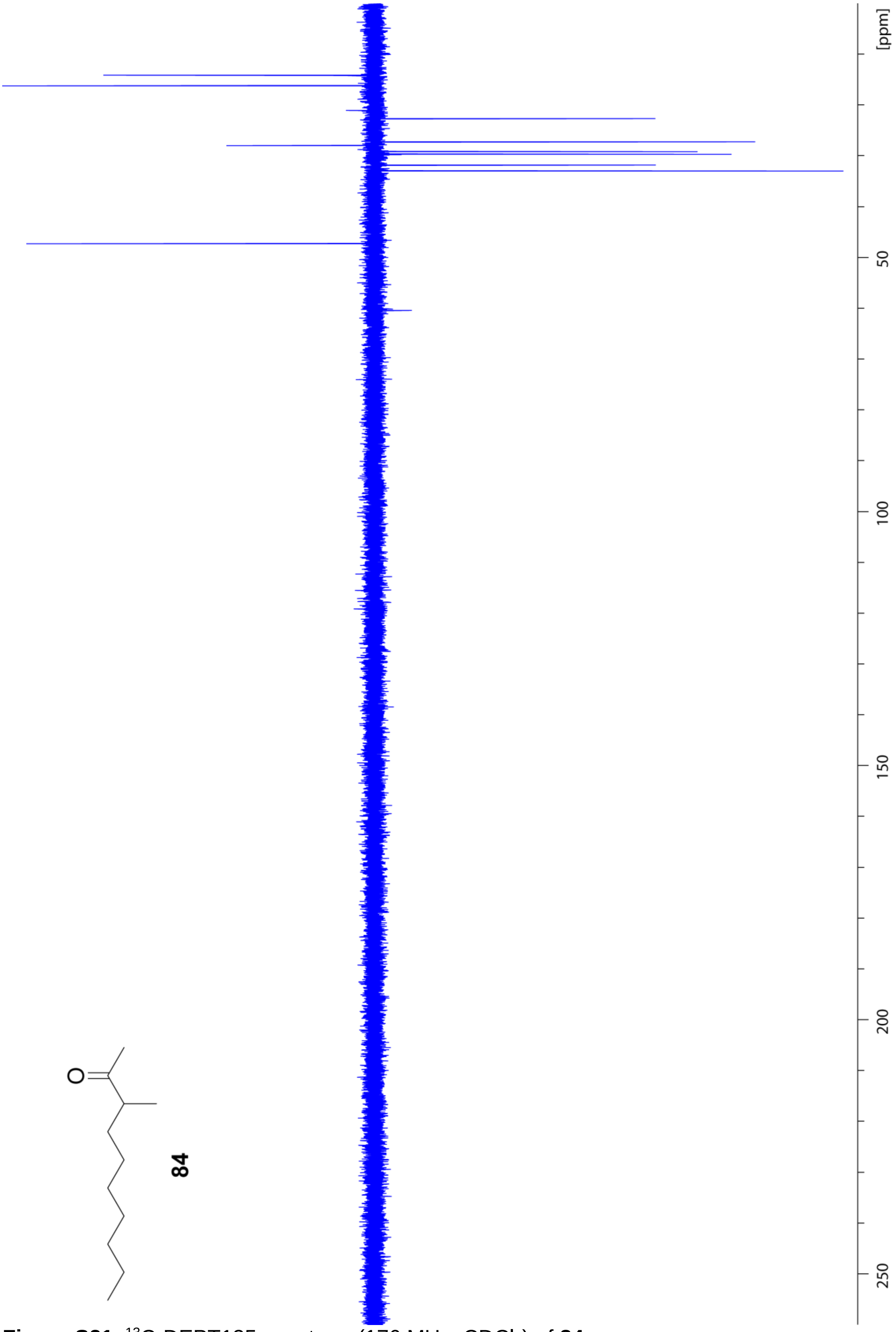

Figure S21. ^{13}C-DEPT135 spectrum (176 MHz, $CDCl_3$) of **84**.

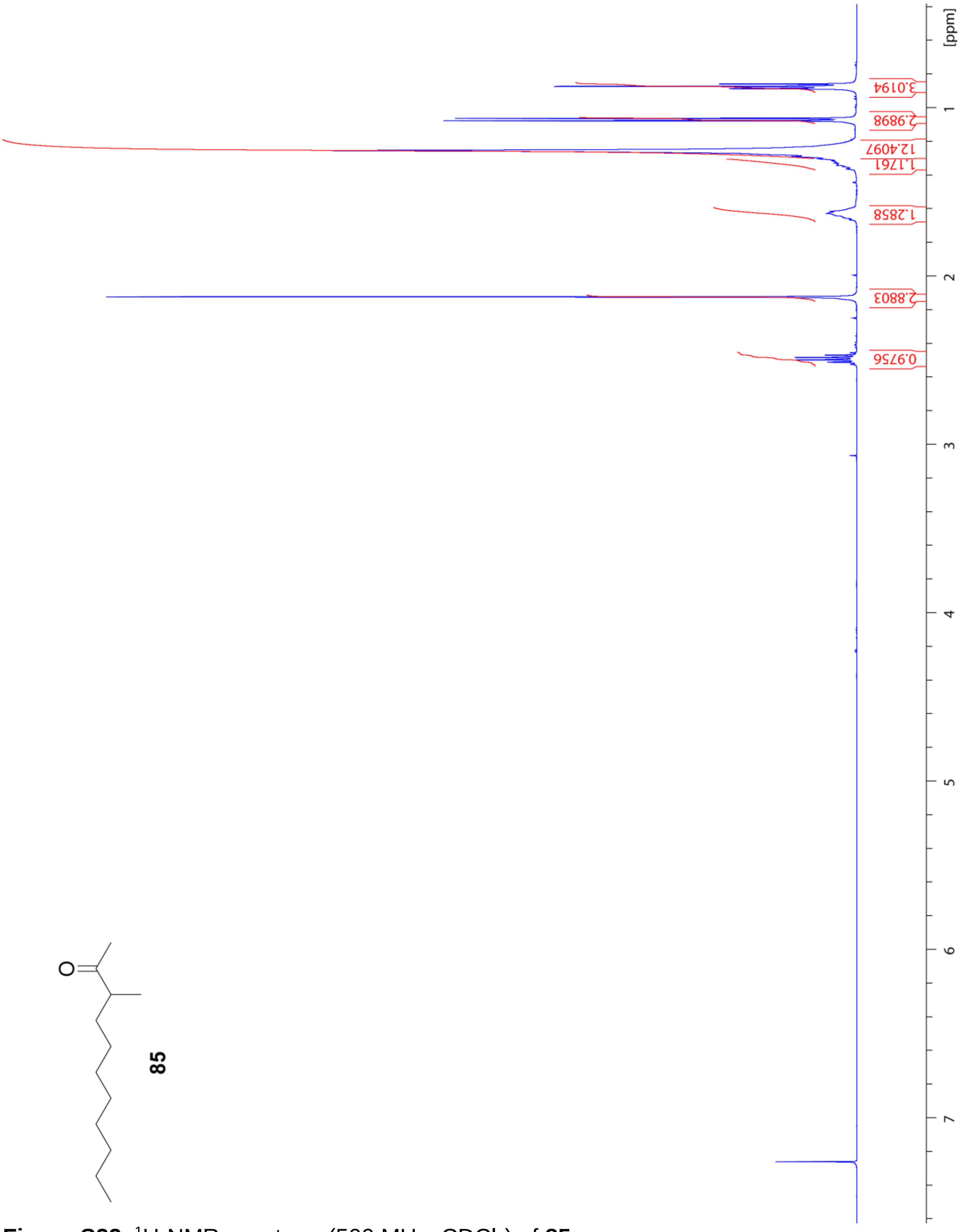

Figure S22. ^{1}H-NMR spectrum (500 MHz, $CDCl_3$) of **85**.

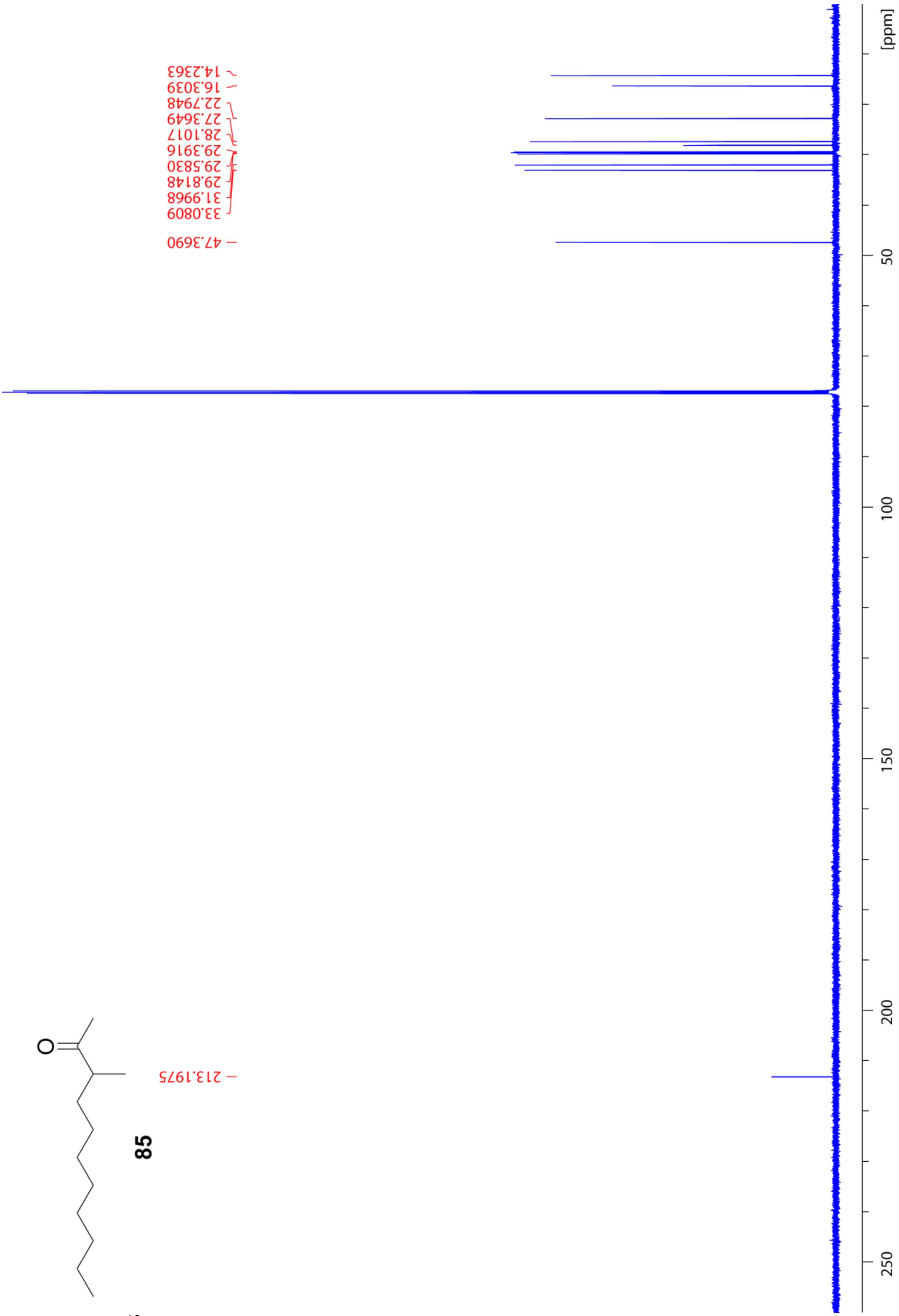

Figure S23. ^{13}C-NMR spectrum (126 MHz, $CDCl_3$) of **85**.

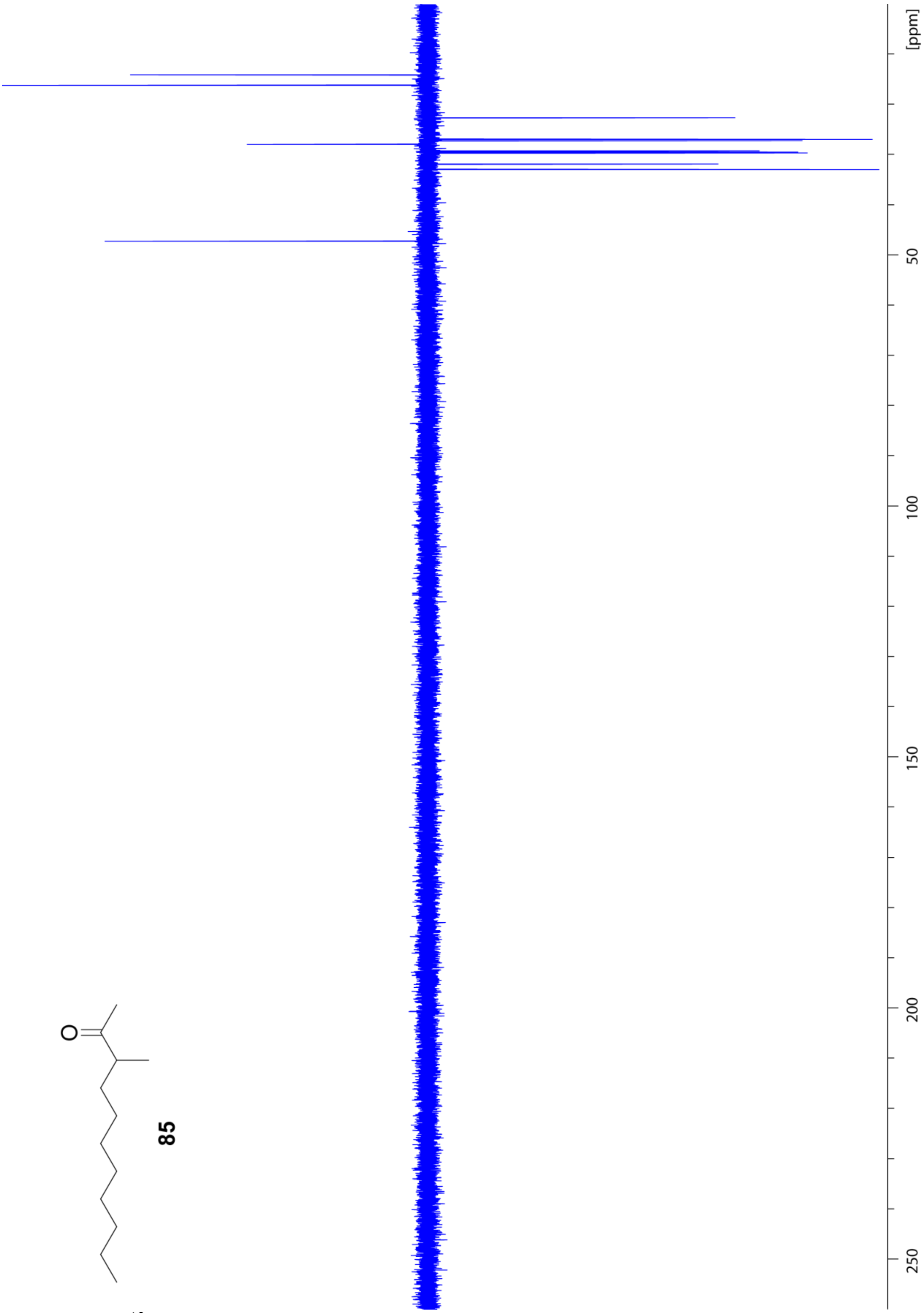

Figure S24. ^{13}C-DEPT spectrum (126 MHz, $CDCl_3$) of **85**.

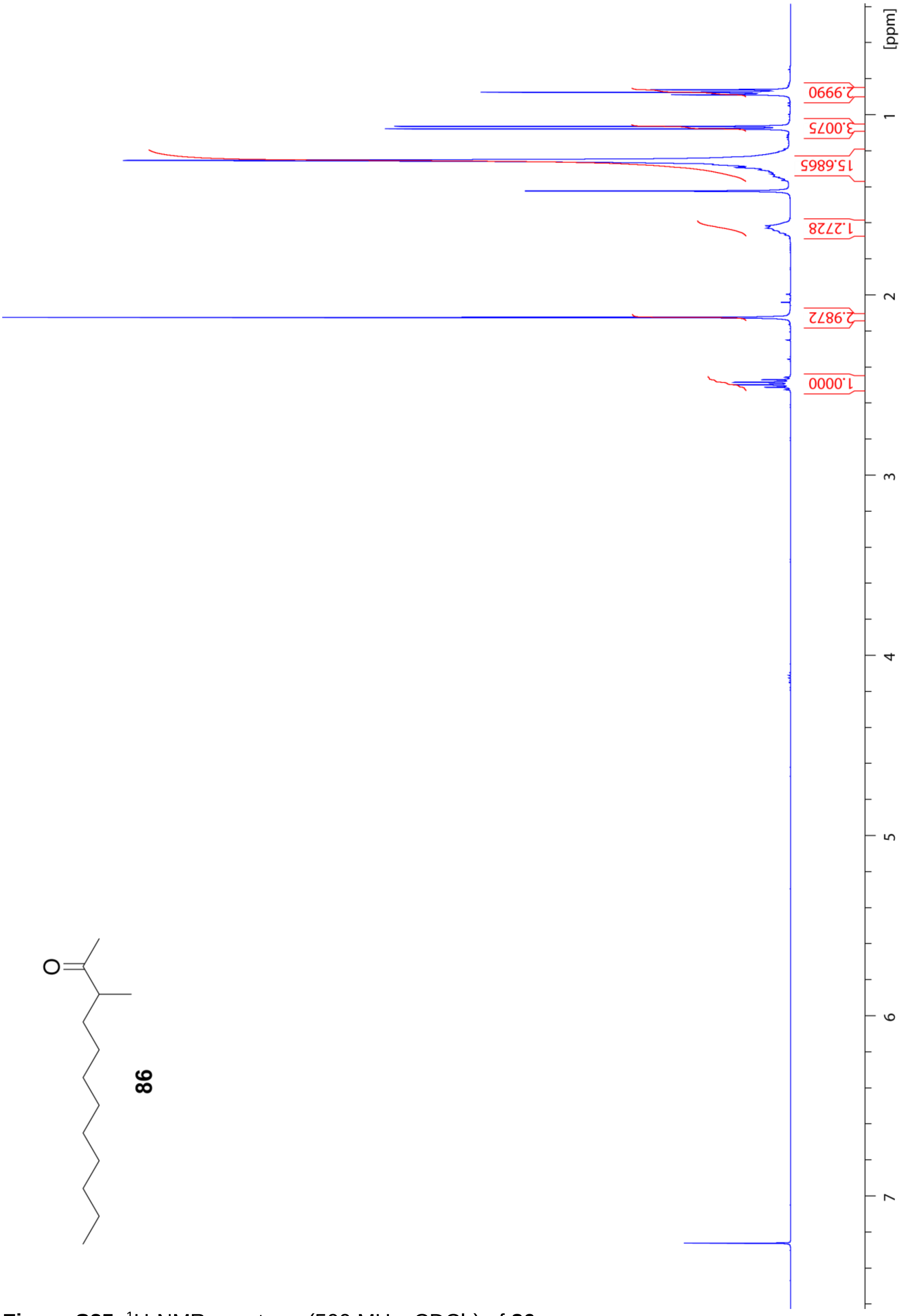

Figure S25. ^{1}H-NMR spectrum (500 MHz, $CDCl_3$) of **86**.

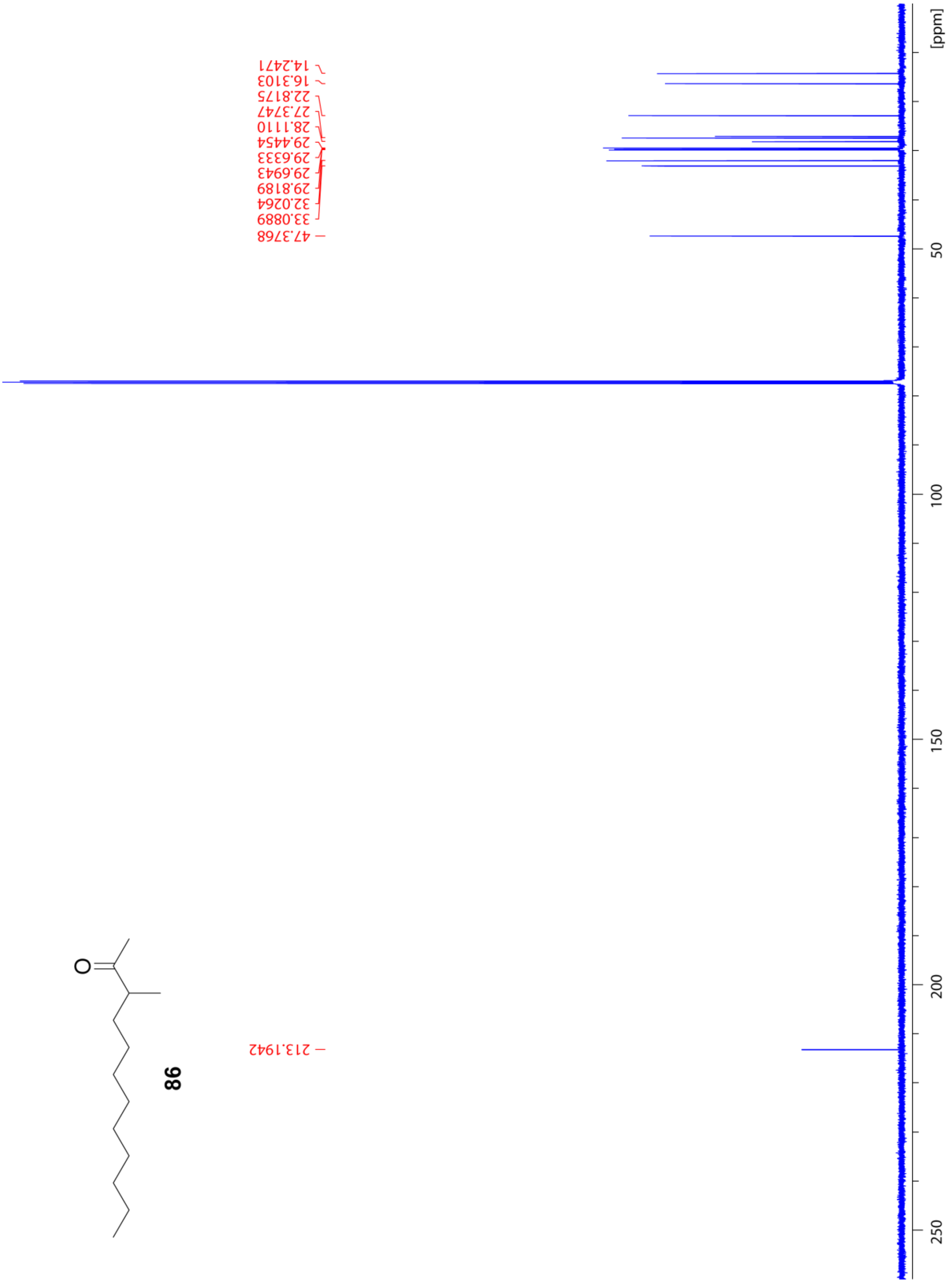

Figure S26. ^{13}C-NMR spectrum (126 MHz, $CDCl_3$) of **86**.

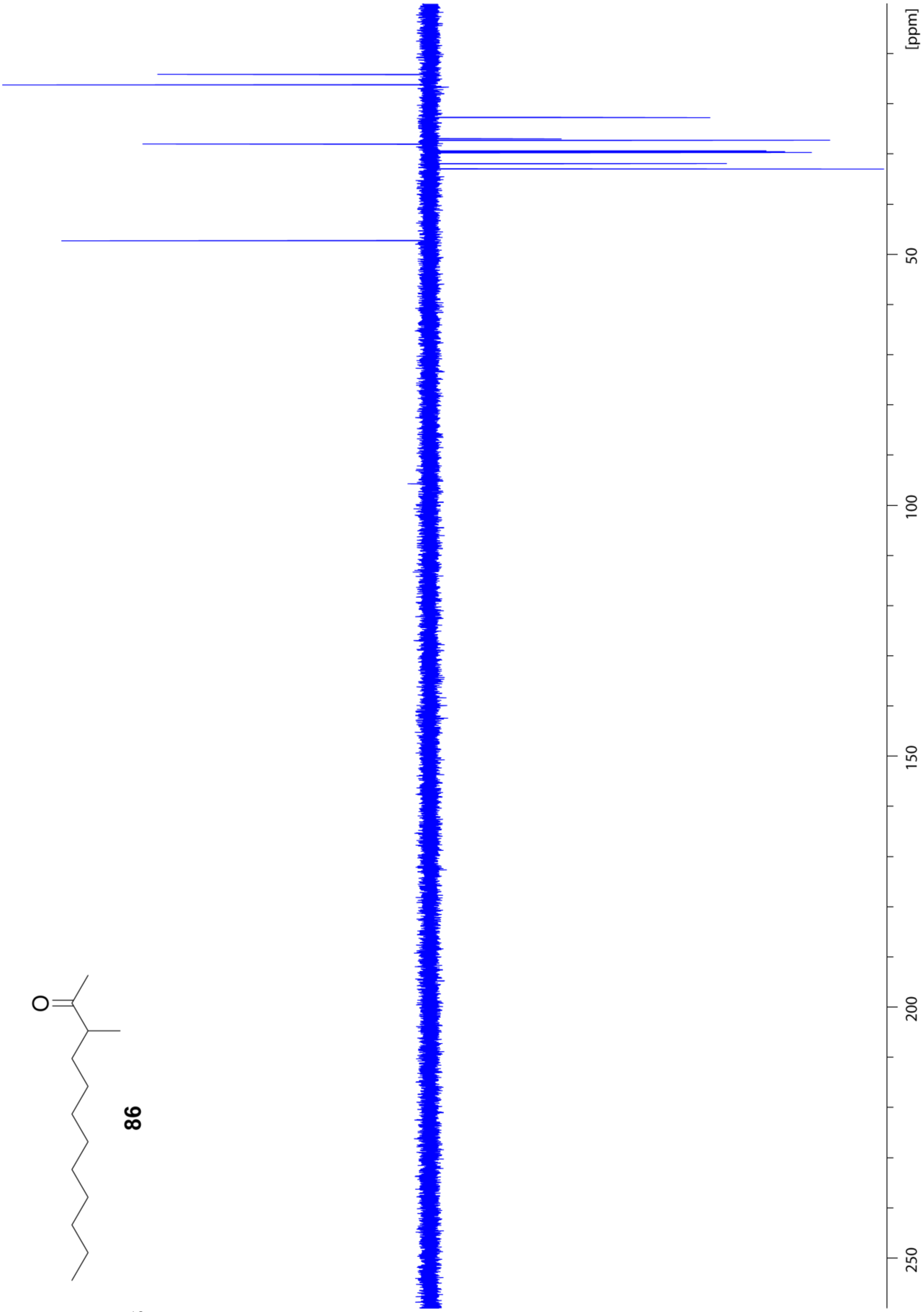

Figure S27. ^{13}C-DEPT135 spectrum (126 MHz, $CDCl_3$) of **86**.

[1] L. Methven, M. Tsoukka, M. J. Oruna-Concha, J. K. Parker, D. S. Mottram, *J. Agric. Food. Chem.* **2007**, *55*, 1427.
[2] H. Rembold, P. Wallner, S. Nitz, H. Kollmannsberger, F. Drawert, *J. Agric. Food Chem.* **1989**, *37*, 659.
[3] R. P. Adams, *Identification of Essential Oil Components by Gas Chromatography/Mass Spectrometry*, *Vol. 4*, Allured, Carol Stream, **2009**.
[4] J. S. Dickschat, S. Wickel, C. J. Bolten, T. Nawrath, S. Schulz, C. Wittmann, *Eur. J. Org. Chem.* **2010**, 2687.
[5] J. S. Dickschat, S. C. Wenzel, H. B. Bode, R. Müller, S. Schulz, *ChemBioChem* **2004**, *5*, 778.
[6] J. Lazarevic, N. Radulovic, R. Palic, B. Zlatkovic, *J. Essential Oil. Res.* **2010**, *22*, 153.
[7] A. D. Beal, D. S. Mottram, *J. Agric. Food Chem.* **1994**, *42*, 2880.
[8] B. M. Harrisin, F. G. Priest, *J. Agric. Food Chem.* **2009**, *57*, 2385.
[9] N. R. Andriamaharavo, Retention Data, NIST Mass Spectrometry Data Center, **2014**.
[10] T. Nawrath, G. F. Mgode, B. Weetjens, S. H. E. Kaufmann, S. Schulz, *Beilstein J. Org. Chem.* **2012**, *8*, 290.
[11] F. Begnaud, C. Pérès, J.-L. Berdagué, *Int. J. Environ. Anal. Chem.* **2003**, *83*, 837.
[13] J. S. Dickschat, T. Martens, T. Brinkhoff, M. Simon, S. Schulz, *Chem. Biodivers.* **2005**, *2*, 837.
[12] W. Haberer, T. Schmidt, P. Schreier, A.-K. Eggert, J. K. Müller, *J. Chem. Ecol.* **2017**, *43*, 971.
[14] R. Splivallo, S. Bossi, M. Maffei, P. Bonfante, *Phytochemistry* **2007**, *68*, 2584.
[15] I. Jerković, G. Hegić, Z. Marijanović, D. Bubalo, *Molecules* **2010**, *15*, 2911.
[16] J. S. Dickschat, E. Helmke, S. Schulz, *Chem. Biodivers.* **2005**, *2*, 318.
[17] C. E. Rostad, W. E. Pereira, *J. High Res. Chromatogr.* **1986**, *9*, 328.
